# Batch effects and the effective design of single-cell gene expression studies

**DOI:** 10.1038/srep39921

**Published:** 2017-01-03

**Authors:** Po-Yuan Tung, John D. Blischak, Chiaowen Joyce Hsiao, David A. Knowles, Jonathan E. Burnett, Jonathan K. Pritchard, Yoav Gilad

**Affiliations:** 1 Department of Human Genetics, University of Chicago, Chicago, Illinois, USA; 2 Committee on Genetics, Genomics, and Systems Biology, University of Chicago, Chicago, Illinois, USA; 3 Department of Genetics, Stanford University, Stanford, CA, USA; 4 Department of Radiology, Stanford University, Stanford, CA, USA; 5 Department of Biology, Stanford University, Stanford, CA, USA; 6 Howard Hughes Medical Institute, Stanford University, CA, USA; 7 Department of Medicine, University of Chicago, Chicago, Illinois, USA

## Abstract

Single-cell RNA sequencing (scRNA-seq) can be used to characterize variation in gene expression levels at high resolution. However, the sources of experimental noise in scRNA-seq are not yet well understood. We investigated the technical variation associated with sample processing using the single-cell Fluidigm C1 platform. To do so, we processed three C1 replicates from three human induced pluripotent stem cell (iPSC) lines. We added unique molecular identifiers (UMIs) to all samples, to account for amplification bias. We found that the major source of variation in the gene expression data was driven by genotype, but we also observed substantial variation between the technical replicates. We observed that the conversion of reads to molecules using the UMIs was impacted by both biological and technical variation, indicating that UMI counts are not an unbiased estimator of gene expression levels. Based on our results, we suggest a framework for effective scRNA-seq studies.

Single-cell genomic technologies can be used to study the regulation of gene expression at unprecedented resolution^1^,^2^. Using single-cell gene expression data, we can begin to effectively characterize and classify individual cell types and cell states, develop a better understanding of gene regulatory threshold effects in response to treatments or stress, and address a large number of outstanding questions that pertain to the regulation of noise and robustness of gene expression programs. Indeed, single cell gene expression data have already been used to study and provide unique insight into a wide range of research topics, including differentiation and tissue development^3^,^4^,^5^, the innate immune response^6^,^7^, and pharmacogenomics^8^,^9^.

Yet, there are a number of outstanding challenges that arose in parallel with the application of single cell technology^10^. A fundamental difficulty, for instance, is the presence of inevitable technical variability introduced during sample processing steps, including but not limited to the conditions of mRNA capture from a single cell, amplification bias, sequencing depth, and variation in pipetting accuracy. These (and other sources of error) may not be unique to single cell technologies, but in the context of studies where each sample corresponds to a single cell, and is thus processed as a single unrepeatable batch, these technical considerations make the analysis of biological variability across single cells particularly challenging.

To better account for technical variability in scRNA-seq experiments, it has become common to add spike-in RNA standards of known abundance to the endogenous samples^11^,^12^. The most commonly used spike-in was developed by the External RNA Controls Consortium (ERCC)^13^; comprising of a set of 96 RNA controls of varying length and GC content. A number of single cell studies focusing on analyzing technical variability based on ERCC spike-in controls have been reported^11^,^12^,^14^,^15^. However, one principle problem with spike-ins is that they do not ‘experience’ all processing steps that the endogenous sample is subjected to. For that reason, it is unknown to what extent the spike-ins can faithfully reflect the error that is being accumulated during the entire sample processing procedure, either within or across batches. In particular, amplification bias, which is assumed to be gene-specific, cannot be addressed by spike-in normalization approaches.

To address challenges related to the efficiency and uniformity with which mRNA molecules are amplified and sequenced in single cells, unique molecule identifiers (UMIs) were introduced to single cell sample processing^16^,^17^,^18^,^19^. The rationale is that by counting molecules rather than the number of amplified sequencing reads, one can account for biases related to amplification, and obtain more accurate estimates of gene expression levels^7^,^12^,^20^. It is assumed that most sources of variation in single cell gene expression studies can be accounted for by using the combination of UMIs and a spike-in based standardization^15^,^20^. Nevertheless, though molecule counts, as opposed to sequencing read counts, are associated with substantially reduced levels of technical variability, a non-negligible proportion of experimental error remains unexplained.

There are a few common platforms in use for scRNA-seq. The automated C1 microfluidic platform (Fluidigm), while more expensive per sample, has been shown to confer several advantages over platforms that make use of droplets to capture single cells^3^,^21^. In particular, smaller samples can be processed using the C1 (when cell numbers are limiting), and the C1 capture efficiency of genes (and RNA molecules) is markedly higher. Notably, in the context of this study, the C1 system also allows for direct confirmation of single cell capture events, in contrast to most other microfluidic-based approaches^3^,^22^. One of the biggest limitations of using the C1 system, however, is that single cell capture and preparation from different conditions are fully independent^23^. Consequently, multiple replicates of C1 collections from the same biological condition are necessary to facilitate estimation of technical variability even with the presence of ERCC spike-in controls^10^. To our knowledge, to date, no study has been purposely conducted to assess the technical variability across batches on the C1 platform.

To address this gap, we collected scRNA-seq data from induced pluripotent stem cell (iPSC) lines of three Yoruba individuals (abbreviation: YRI) using C1 microfluidic plates. Specifically, we performed three independent C1 collections per each individual to disentangle batch effects from the biological covariate of interest, which, in this case, is the difference between individuals. Both ERCC spike-in controls and UMIs were included in our sample processing. With these data, we were able to elucidate technical variability both within and between C1 batches and thus provide a deep characterization of cell-to-cell variation in gene expression levels across individuals.

## Results

### Study design and quality control

**Figure 1:**
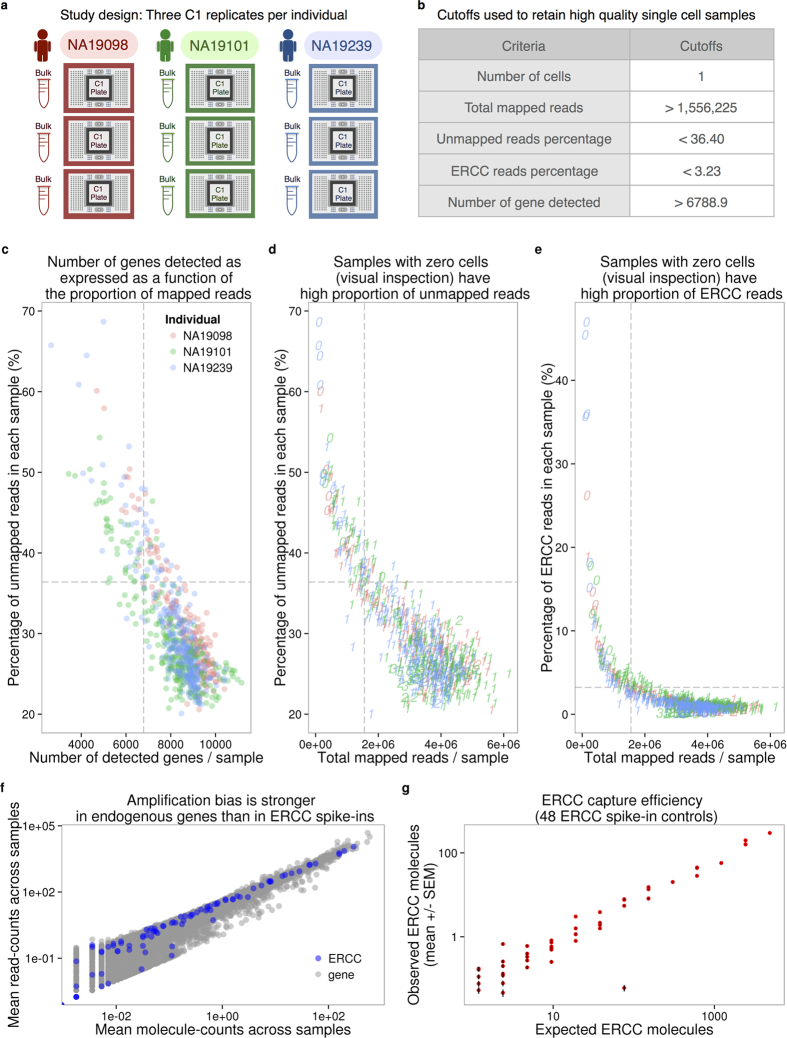
Experimental design and quality control of scRNA-seq. (**a**) Three C1 96 well-integrated fluidic circuit (IFC) replicates were collected from each of the three Yoruba individuals. A bulk sample was included in each batch. (**b**) Summary of the cutoffs used to remove data from low quality cells that might be ruptured or dead (See Supplementary Fig. S1 for details). (**c–e**) To assess the quality of the scRNA-seq data, the capture efficiency of cells and the faithfulness of mRNA fraction amplification were determined based on the proportion of unmapped reads, the number of detected genes, the numbers of total mapped reads, and the proportion of ERCC spike-in reads across cells. The dash lines indicate the cutoffs summarized in panel (**b**). The three colors represent the three individuals (NA19098 in red, NA19101 in green, and NA19239 in blue), and the numbers indicate the cell numbers observed in each capture site on C1 plate. (**f**) Scatterplots in log scale showing the mean read counts and the mean molecule counts of each endogenous gene (grey) and ERCC spike-ins (blue) from the 564 high quality single cell samples before removal of genes with low expression. (**g**) mRNA capture efficiency shown as observed molecule count versus number of molecules added to each sample, only including the 48 ERCC spike-in controls remaining after removal of genes with low abundance. Each red dot represents the mean +/− SEM of an ERCC spike-in across the 564 high quality single cell samples.

We collected single cell RNA-seq (scRNA-seq) data from three YRI iPSC lines using the Fluidigm C1 microfluidic system followed by sequencing. We added ERCC spike-in controls to each sample, and used 5-bp random sequence UMIs to allow for the direct quantification of mRNA molecule numbers. For each of the YRI lines, we performed three independent C1 collections; each replicate was accompanied by processing of a matching bulk sample using the same reagents. This study design (Fig. 1a and Supplementary Table S1) allows us to estimate error and variability associated with the technical processing of the samples, independently from the biological variation across single cells of different individuals. We were also able to estimate how well scRNA-seq data can recapitulate the RNA-seq results from population bulk samples.

In what follows, we describe data as originating from different samples when we refer to data from distinct wells of each C1 collection. Generally, each sample corresponds to a single cell. In turn, we describe data as originating from different replicates when we refer to all samples from a given C1 collection, and from different individuals when we refer to data from all samples and replicates of a given genetically distinct iPSC line.

We obtained an average of 6.3 +/− 2.1 million sequencing reads per sample (range 0.4–11.2 million reads). We processed the sequencing reads using a standard alignment approach (see Methods) and performed multiple quality control analyses. As a first step, we estimated the proportion of ERCC spike-in reads from each sample. We found that, across samples, sequencing reads from practically all samples of the second replicate of individual NA19098 included unusually high ERCC content compared to all other samples and replicates (Supplementary Fig. S1). We concluded that a pipetting error led to excess ERCC content in this replicate and we excluded the data from all samples of this replicate in subsequent analyses. With the exception of the excluded samples, data from all other replicates seem to have similar global properties (using general metrics; Fig. 1c–e and Supplementary Fig. S1).

We next examined the assumption that data from each sample correspond to data from a single cell. After the cell sorting was complete, but before the processing of the samples, we performed visual inspection of the C1 microfluidic plates. Based on that visual inspection, we flagged 21 samples that did not contain any cell, and 54 samples that contained more than one cell (across all batches). Visual inspection of the C1 microfluidic plate is an important quality control step, but it is not infallible. We therefore filtered data from the remaining samples based on the number of total mapped reads, the percentage of unmapped reads, the percentage of ERCC spike-in reads, and the number of genes detected (Fig. 1b–e). We chose data-driven inclusion cutoffs for each metric, based on the 95th percentile of the respective distributions for the 21 libraries that were amplified from samples that did not include a cell based on visual inspection (Supplementary Fig. S1). Using this approach, we identified and removed data from 15 additional samples that were classified as originating from a single cell based on visual inspection, but whose data were more consistent with a multiple-cell origin based on the number of total molecules, the concentration of cDNA amplicons, and the read-to-molecule conversion efficiency (defined as the number of total molecules divided by the number of total reads; Supplementary Fig. S2). At the conclusion of these quality control analyses and exclusion steps, we retained data from 564 high quality samples, which correspond, with reasonable confidence, to 564 single cells, across eight replicates from three individuals (Supplementary Table S2).

Our final quality check focused on the different properties of sequencing read and molecule count data. We considered data from the 564 high quality samples and compared gene specific counts of sequencing read and molecules. We found that while gene-specific reads and molecule counts are exceptionally highly correlated when we considered the ERCC spike-in data (r = 0.99; Fig. 1f), these counts are somewhat less correlated when data from the endogenous genes are considered (r = 0.92). Moreover, the gene-specific read and molecule counts correlation is noticeably lower for genes that are expressed at lower levels (Fig. 1f). These observations concur with previous studies^12^,^20^ as they underscore the importance of using UMIs in single cell gene expression studies.

**Figure 2:**
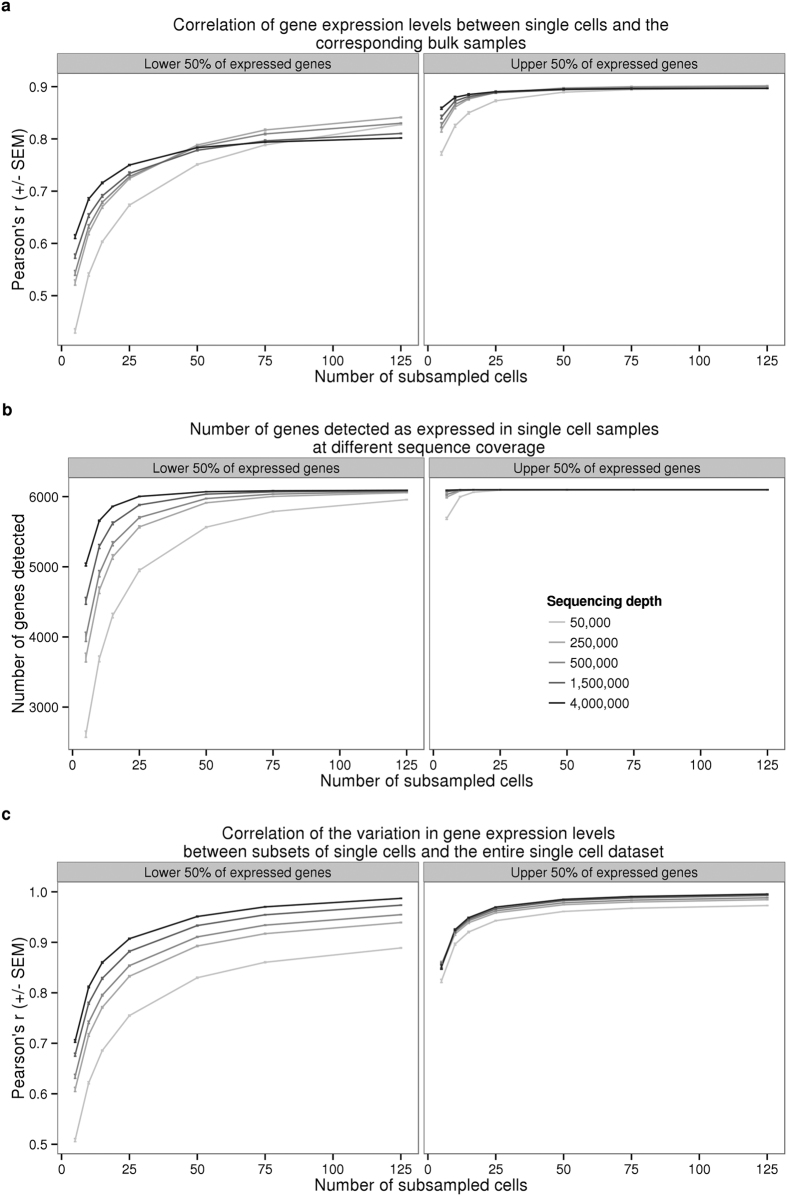
The effect of sequencing depth and cell number on single cell UMI estimates. Sequencing reads from all the high quality single cells collected for NA19239 were subsampled to the indicated sequencing depth and cell number, and subsequently converted to molecules using the UMIs. Each point represents the mean +/− SEM of 10 random draws of the indicated cell number. The left panel displays the results for 6,097 (50% of detected) genes with lower expression levels and the right panel the results for 6,097 genes with higher expression levels. (**a**) Pearson correlation of aggregated gene expression level estimates from single cells compared to the bulk sequencing samples. (**b**) Total number of genes detected with at least one molecule in at least one of the single cells. (**c**) Pearson correlation of cell-to-cell gene expression variance estimates from subsets of single cells compared to the full single cell data set.

We proceeded by investigating the effect of sequencing depth and the number of single cells collected on multiple properties of the data. To this end, we repeatedly subsampled single cells and sequencing reads to assess the correlation of the single cell gene expression estimates to the bulk samples, the number of genes detected, and the correlation of the cell-to-cell gene expression variance estimates between the reduced subsampled data and the full single cell gene expression data set (Fig. 2). We observed quickly diminishing improvement in all three properties with increasing sequencing depth and the number of sampled cells, especially for highly expressed genes. For example, a per cell sequencing depth of 1.5 million reads (which corresponds to ~50,000 molecules) from each of 75 single cells was sufficient for effectively quantifying even the lower 50% of expressed genes. To be precise, at this level of subsampling for individual NA19239, we were able to detect a mean of 6068 genes out of 6097 genes expressed in the bulk samples (the bottom 50%; Fig. 2b); the estimated single cell expression levels of these genes (summed across all cells) correlated with the bulk sample gene expression levels with a mean Pearson coefficient of 0.8 (Fig. 2a), and the estimated cell-to-cell variation in gene expression levels was correlated with the variation estimated from the full data set with a mean Pearson coefficient of 0.95 (Fig. 2c). We observed similar results when examining each batch separately (Supplementary Fig. S3).

### Batch effects associated with UMI-based single cell data

In the context of the C1 platform, typical study designs make use of a single C1 plate (batch/replicate) per biological condition. In that case, it is impossible to distinguish between biological and technical effects associated with the independent capturing and sequencing of each C1 replicate. We designed our study with multiple technical replicates per biological condition (individual) in order to directly and explicitly estimate the batch effect associated with independent C1 preparations (Fig. 1a).

As a first step in exploring batch effects, we examined the gene expression profiles across all single cells that passed our quality checks (as reported above) using raw molecule counts (without standardization). Using principal component analysis (PCA) for visualization, we observed – as expected - that the major source of variation in data from single cells is the individual origin of the sample. Specifically, we found that the proportion of variance due to individual was larger (median: 8%) than variance due to C1 batch (median: 4%; Kruskal-Wallis test; *P* < 0.001, Supplementary Fig. S4; see Methods for details of the variance component analysis). Yet, variation due to C1 batch is also substantial - data from single cell samples within a batch are more correlated than that from single cells from the same individual but different batches (Kruskal-Wallis test; *P* < 0.001).

**Figure 3:**
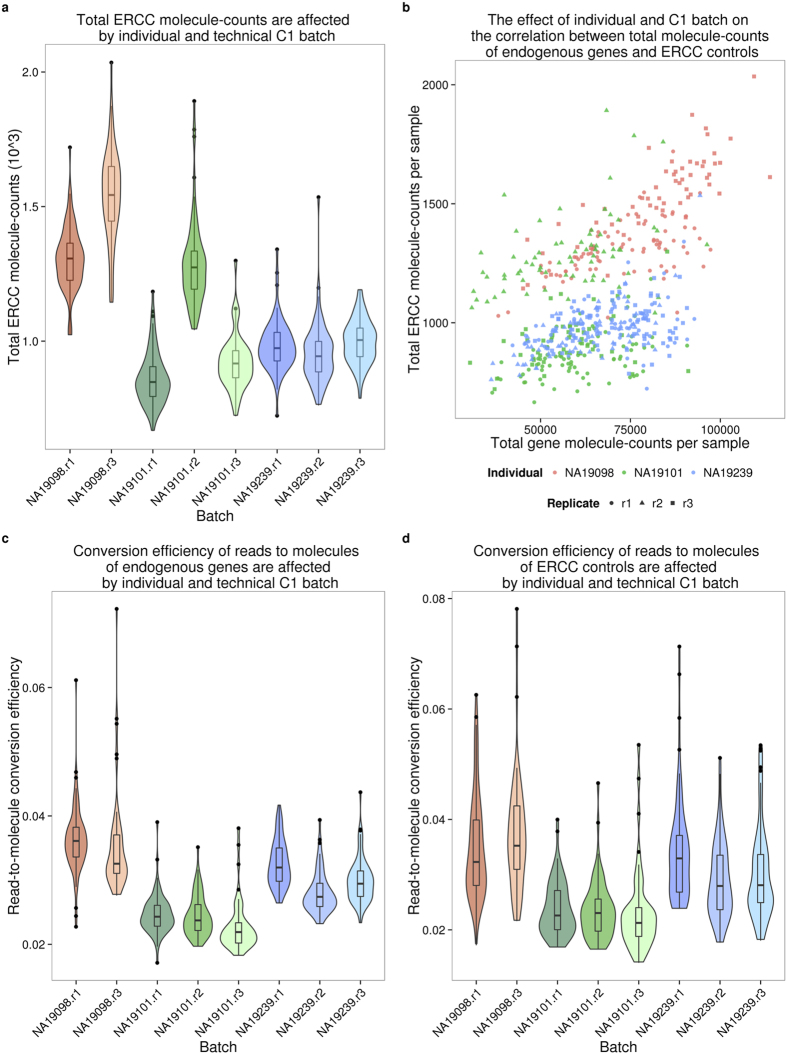
Batch effect of scRNA-seq data using the C1 platform. (**a**) Violin plots of the number of total ERCC spike-in molecule-counts in single cell samples per C1 replicate. (**b**) Scatterplot of the total ERCC molecule-counts and total gene molecule-counts. The colors represent the three individuals (NA19098 is in red, NA19101 in green, and NA19239 in blue). Data from different C1 replicates is plotted in different shapes. (**c** and **d**) Violin plots of the reads to molecule conversion efficiency (total molecule-counts divided by total read-counts per single cells) by C1 replicate. The endogenous genes and the ERCC spike-ins are shown separately in (**c**) and (**d**), respectively. There is significant difference across individuals of both endogenous genes (*P* < 0.001) and ERCC spike-ins (*P* < 0.05). The differences across C1 replicates per individual of endogenous genes and ERCC spike-ins were also evaluated (both *P* < 0.01).

Could we account for the observed batch effects using the ERCC spike-in controls? In theory, if the total ERCC molecule-counts are affected only by technical variability, the spike-ins could be used to correct for batch effects even in a study design that entirely confounds biological samples with C1 preparations. To examine this, we first considered the relationship between total ERCC molecule-counts and total endogenous molecule-counts per sample. If only technical variability affects ERCC molecule-counts, we expect the technical variation in the spike-ins (namely, variation between C1 batches) to be consistent, regardless of the individual assignment. Indeed, we observed that total ERCC molecule-counts are significantly different between C1 batches (F-test; *P* < 0.001). However, total ERCC molecule-counts are also quite different across individuals, when variation between batches is taken into account (LRT; *P* = 0.08; Fig. 3a). This observation suggests that both technical and biological variation affect total ERCC molecule-counts. In addition, while we observed a positive relationship between total ERCC molecule-counts and total endogenous molecule-counts per sample, this correlation pattern differed across C1 batches and across individuals (F-test; *P* < 0.001; Fig. 3b).

To more carefully examine the technical and biological variation of ERCC spike-in controls, we assessed the ERCC per-gene expression profile. We observed that the ERCC gene expression data from samples of the same batch were more correlated than data from samples across batches (Kruskal-Wallis test; Chi-squared *P* < 0.001). However, the proportion of variance explained by the individual was significantly larger than the variance due to C1 batch (median: 9% vs. 5%, Chi-squared test; *P* < 0.001, Supplementary Fig. S4), lending further support to the notion that biological variation affects the ERCC spike in data. Based on these analyses, we concluded that ERCC spike-in controls cannot be used to effectively account for the batch effect associated with independent C1 preparations.

We explored additional properties in the data that may be associated with a batch effect. To do so, we focused on the read-to-molecule conversion rates, i.e. the rates at which sequencing reads are converted to molecule counts based on the UMI sequences. We defined read-to-molecule conversion efficiency as the total molecule-counts divided by the total reads-counts in each sample, considering separately the reads/molecules that correspond to endogenous genes or ERCC spike-ins (Fig. 3c and d). We observed a significant batch effect in the read-to-molecule conversion efficiency of both ERCC (F-test; *P* < 0.05) and endogenous genes (F-test; *P* < 0.001) across C1 replicates from the same individual. Moreover, the difference in read-to-molecule conversion efficiency across the three individuals was significant not only for endogenous genes (LRT; *P* < 0.01, Fig. 3c) but also in the ERCC spike-ins (LRT; *P* < 0.01, Fig. 3d).

### Measuring regulatory noise in single-cell gene expression data

Our analysis indicated that there is a considerable batch effect in the single cell gene expression data collected from the C1 platform. We thus sought an approach that would account for the batch effect and allow us to study biological properties of the single-cell molecule count-based estimates of gene expression levels, albeit in a small sample of just three individuals. As a first step, we adjusted the raw molecule counts by using a Poisson approximation to account for the random use of identical UMI sequences in molecules from highly expressed genes (this was previously termed a correction for the UMI ‘collision probability’^17^). We then excluded data from genes whose inferred molecule count exceeded 1,024 (the theoretical number of UMI sequences) – this step resulted in the exclusion of data from 6 mitochondrial genes.

**Figure 4:**
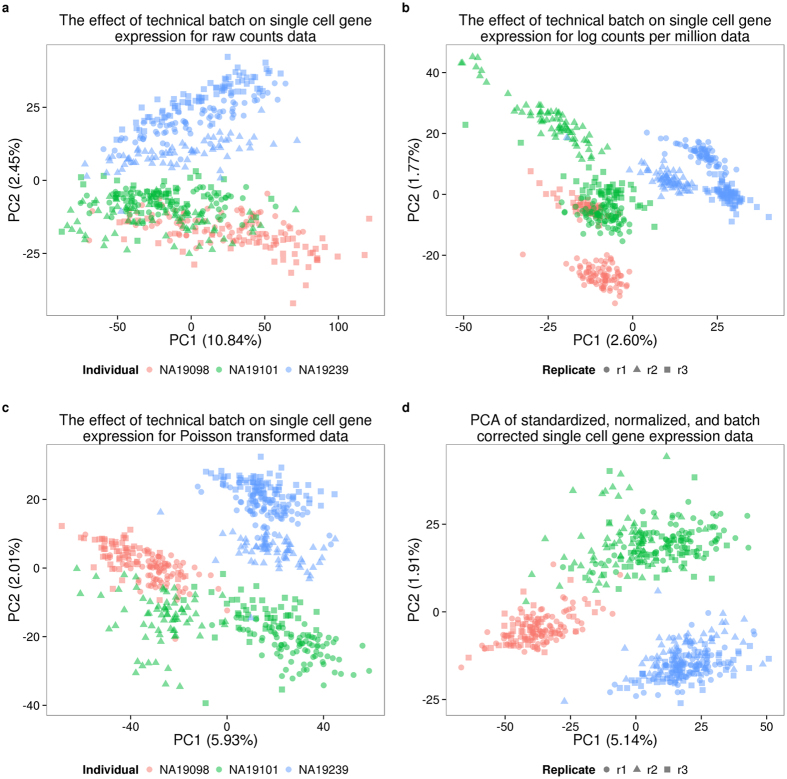
Normalization and removal of technical variability. Principal component (PC) 1 versus PC2 of the (**a**) raw molecule counts, (**b**) log_2_ counts per million (cpm), (**c**) Poisson transformed expression levels (accounting for technical variability modeled by the ERCC spike-ins), and (**d**) batch-corrected expression levels. The colors represent the three individuals (NA19098 in red, NA19101 in green, and NA19239 in blue). Data from different C1 replicates is plotted in different shapes.

We next incorporated a standardization step by computing log transformed counts-per-million (cpm) to remove the effect of different sequencing depths, as is the common practice for the analysis of bulk RNA-seq data (Fig. 4a and b). We used a Poisson generalized linear model to normalize the endogenous molecule log_2_ cpm values by the observed molecule counts of ERCC spike-ins across samples. While we do not expect this step to account for the batch effect (as discussed above), we reasoned that the spike-ins allow us to account for a subset of technical differences between samples, for example, those that arise from differences in RNA concentration (Fig. 4c).

Finally, to account for the technical batch effect, we modeled between-sample correlations in gene expression within C1 replicates (see Methods). Our approach is similar in principle to limma, which was initially developed for adjusting within-replicate correlations in microarray data^24^. We assume that samples within each C1 replicate share a component of technical variation, which is independent of biological variation across individuals. We fit a linear mixed model for each gene, which includes a fixed effect for individual and a random effect for batch. The batch effect is specific to each C1 replicate, and is independent of biological variation across individuals. We use this approach to estimate and remove the batch effect associated with different C1 preparations (Fig. 4d).

Once we removed the unwanted technical variability, we focused on analyzing biological variation in gene expression between single cells. Our goal was to identify inter-individual differences in the amount of variation in gene expression levels across single cells, or in other words, to identify differences between individuals in the amount of regulatory noise^25^. In this context, regulatory noise is generally defined as the coefficient of variation (CV) of the gene expression levels of single cells^26^. In the following, we used the standardized, normalized, batch-corrected molecule count gene expression data to estimate regulatory noise (Fig. 4d). To account for heteroscedasticity from Poisson sampling, we adjusted the CV values by the average gene-specific expression level across cells of the same individual. The adjusted CV is robust both to differences in gene expression levels, as well as to the proportion of gene dropouts in single cells.

To investigate the effects of gene dropouts (the lack of molecule representation of an expressed gene^6^,^11^) on our estimates of gene expression noise, we considered the association between the proportion of cells in which a given gene is undetected (namely, the gene-specific dropout rate), the average gene expression level, and estimates of gene expression noise. Across all genes, the median gene-specific dropout was 22 percent. We found significant individual differences (LRT; *P* < 10^−5^) in gene-specific dropout rates between individuals in more than 10% (1,214 of 13,058) of expressed endogenous genes. As expected, the expression levels, and the estimated variation in expression levels across cells, are both associated with gene-specific dropout rates (Supplementary Fig. S5). However, importantly, adjusted CVs are not associated with dropout rates (Spearman’s correlation = 0.04; Supplementary Fig. S5), indicating that adjusted CV measurements are not confounded by the dynamic range of single-cell gene expression levels.

**Figure 5:**
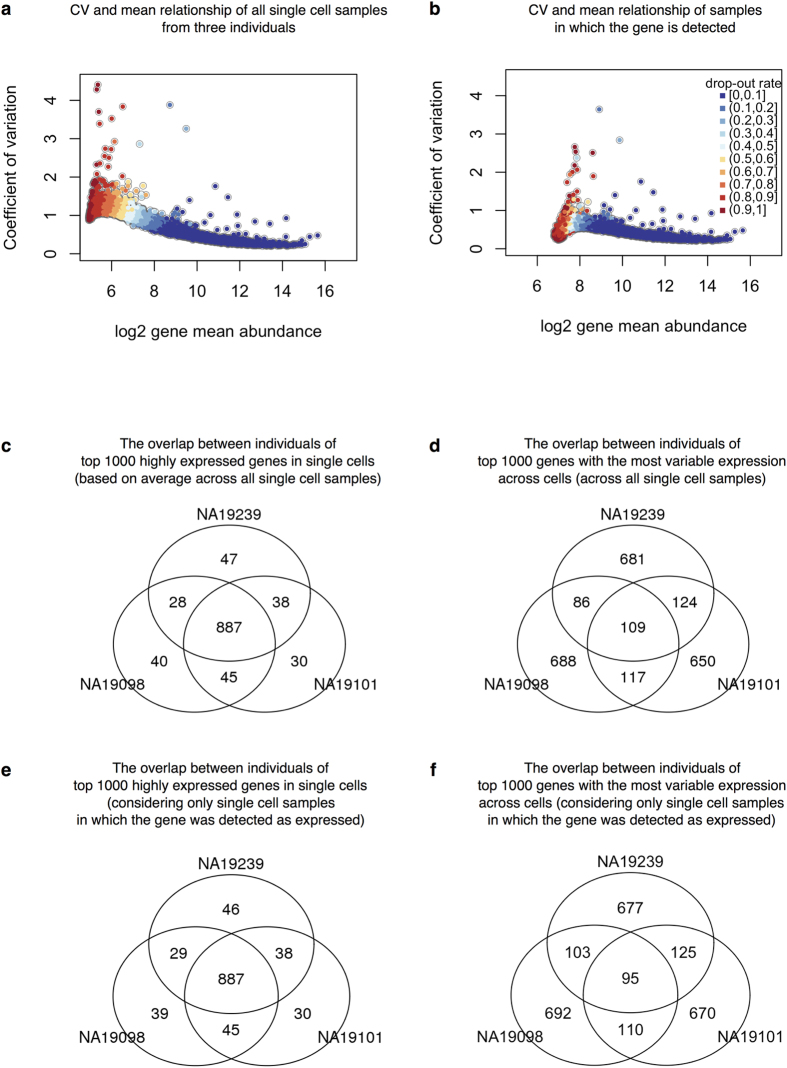
Cell-to-cell variation in gene expression. Adjusted CV plotted against average molecule counts across all cells in (**a**) and across only the cells in which the gene is expressed (**b**), including data from all three individuals. Each dot represents a gene, and the color indicates the corresponding gene-specific dropout rate (the proportion of cells in which the gene is undetected). (**c** and **d**) Venn diagrams showing the overlaps of top 1000 genes across individuals based on mean expression level in (**c**) and based on adjusted CV values in (**d**), considering only the cells in which the gene is expressed. (**e** and **f** ) Similarly, Venn diagrams showing the overlaps of top 1000 genes across individuals based on mean expression level in (**e**) and based on adjusted CV values in (**f** ), across all cells.

We thus estimated mean expression levels and regulatory noise (using adjusted CV) for each gene, by either including (Fig. 5a) or excluding (Fig. 5b) samples in which the gene was not detected/expressed. We first focused on general trends in the data. We ranked genes in each individual by their mean expression level as well as by their estimated level of variation across single cells. When we considered samples in which a gene was expressed, we found that 887 of the 1,000 most highly expressed genes in each individual are common to all three individuals (Fig. 5c). In contrast, only 95 of the 1,000 most highly variable (noisy) genes in each individual were common to all three individuals (Fig. 5d). We found similar results when we considered data from all single cells, regardless of whether the gene was detected as expressed (Fig. 5e and f). In particular, the set of 887 highly expressed genes was also detected as common to all three individuals, while 80% of the 95 most highly variable genes (76 genes) were detected as common to all three individuals when including samples in which the gene was not detected as expressed.

Next, we identified genes whose estimated regulatory noise (based on the adjusted CV) is significantly different between individuals. For the purpose of this analysis, we only included data from cells in which the gene was detected as expressed. Based on permutations (Supplementary Fig. S6), we classified the estimates of regulatory noise of 560 genes as significantly different across individuals (empirical *P* < 0.0001, Supplementary Fig. S7 for examples; Supplementary Table S3 for gene list). These 560 genes are enriched for genes involved in protein translation, protein disassembly, and various biosynthetic processes (Supplementary Table S4). Interestingly, among the genes whose regulatory noise estimates differ between individuals, we found two pluripotency genes, *KLF4* and *DPPA2* (Supplementary Fig. S8).

## Discussion

### Study design and sample size for scRNA-seq

Our nested study design allowed us to explicitly estimate technical batch effects associated with single cell sample processing on the C1 platform. We found previously unreported technical sources of variation associated with the C1 sample processing and the use of UMIs, including the property of batch-specific read-to-molecule conversion efficiency. As we used a well-replicated nested study design, we were able to model, estimate, and account for the batch while maintaining individual differences in gene expression levels. We believe that our observations indicate that future studies should avoid confounding C1 batch and individual source of single cell samples. Instead, we recommend a balanced study design consisting of multiple individuals within a C1 plate and multiple C1 replicates (for example, Supplementary Fig. S9). The origin of each cell can then be identified using the RNA sequencing data. Indeed, using a method originally developed for detecting sample swaps in DNA sequencing experiments^27^, we were able to correctly identify the correct YRI individual of origin for all the single cells from the current experiment by comparing the polymorphisms identified using the RNA-seq reads to the known genotypes for all 120 YRI individuals of the International HapMap Project^28^ (Supplementary Fig. S9). The mixed-individual-plate is an attractive study design because it allows one to account for the batch effect without the requirement to explicitly spend additional resources on purely technical replication (because the total number of cells assayed from each individual can be equal to a design in which one individual is being processed in using a single C1 plate).

We also addressed additional study design properties with respect to the desired number of single cells and the desired depth of sequencing (Fig. 2). Similar assessments have been previously performed for single cell sequencing with the C1 platform without the use of UMIs^21^,^29^, but no previous study has investigated the effects of these parameters for single cells studies using UMIs. We focused on recapitulating the gene expression levels observed in bulk sequencing experiments, detecting as many genes as possible, and accurately measuring the cell-to-cell variation in gene expression levels. We recommend sequencing at least 75 high quality cells per biological condition with a minimum of 1.5 million raw reads per cell to obtain optimal performance of these three metrics.

### The limitations of the ERCC spike-in controls

The ERCC spike-in controls have been used in previous scRNA-seq studies to identify low quality single cell samples, infer the absolute total number of molecules per cell, and model the technical variability across cells^11^,^12^,^14^,^15^. In our experience, the ERCC controls are not particularly well-suited for any one of these tasks, much less all three. With respect to identifying low quality samples, we indeed observed that samples with no visible cell had a higher percentage of reads mapping to the ERCC controls, as expected. However, there was no clear difference between low and high quality samples in the percentage of ERCC reads or molecules, and thus any arbitrarily chosen cutoff would be associated with considerable error (Fig. 1e). With respect to inferring the absolute total number of molecules per cell, we observed that the biological covariate of interest (difference between the three YRI individuals), rather than batch, explained a large proportion of the variance in the ERCC counts (Supplementary Fig. S4), and furthermore that the ERCC controls were also affected by the individual-specific effect on the read-to-molecule conversion rate (Fig. 3d). Thus ERCC-based corrected estimates of total number of molecules per cell, across technical or biological replicates, are expected to be biased. Because the batch effects associated with the ERCC controls are driven by the biological covariate of interest, they will also impede the modeling of the technical variation in single cell experiments that confound batch and the biological source of the single cells.

More generally, it is inherently difficult to model unknown sources of technical variation using so few genes^30^ (only approximately half of the 92 ERCC controls are detected in typical single cell experiments), and the ERCC controls are also strongly impacted by technical sources of variation even in bulk RNA-seq experiments^31^. Lastly, from a theoretical perspective, the ERCC controls have shorter polyA tails and are overall shorter than mammalian mRNAs. For these reasons, we caution against the reliance of ERCC controls in scRNA-seq studies and highlight that an alternative set of controls that more faithfully mimics mammalian mRNAs and provides more detectable spike-in genes is desired. Our recommendation is to include total RNA from a distant species, for example using RNA from *Drosophila melanogaster* in studies of single cells from humans.

### Outlook

Single cell experiments are ideally suited to study gene regulatory noise and robustness^32^,^33^. Yet, in order to study the biological noise in gene expression levels, it is imperative that one should be able to effectively estimate and account for the technical noise in single cell gene expression data. Our results indicate that previous single cells gene expression studies may not have been able to distinguish between the technical and the biological components of variation, because single cell samples from each biological condition were processed on a single C1 batch. When technical noise is properly accounted for, even in this small pilot study, our findings indicate pervasive inter-individual differences in gene regulatory noise, independently of the overall gene expression level.

## Methods

### Ethics statement

The YRI cell lines were purchased from CCR. The original samples were collected by the HapMap project between 2001–2005. All of the samples were collected with extensive community engagement, including discussions with members of the donor communities about the ethical and social implications of human genetic variation research. Donors gave broad consent to future uses of the samples, including their use for extensive genotyping and sequencing, gene expression and proteomics studies, and all other types of genetic variation research, with the data publicly released.

### Cell culture of iPSCs

Undifferentiated feeder-free iPSCs reprogrammed from LCLs of Yoruba individuals in Ibadan, Nigeria (abbreviation: YRI)^28^ were grown in E8 medium (Life Technologies)^34^ on Matrigel-coated tissue culture plates with daily media feeding at 37 °C with 5% (vol/vol) CO2. For standard maintenance, cells were split every 3–4 days using cell release solution (0.5 mM EDTA and NaCl in PBS) at the confluence of roughly 80%. For the single cell suspension, iPSCs were individualized by Accutase Cell Detachment Solution (BD) for 5–7 minutes at 37 °C and washed twice with E8 media immediately before each experiment. Cell viability and cell counts were then measured by the Automated Cell Counter (Bio-Rad) to generate resuspension densities of 2.5 × 105 cells/mL in E8 medium for C1 cell capture.

### Single cell capture and library preparation

Single cell loading and capture were performed following the Fluidigm protocol (PN 100-7168). Briefly, 30 *μ*l of C1 Suspension Reagent was added to a 70-*μ*l aliquot of ~17,500 cells. Five *μ*l of this cell mix were loaded onto 10–17 *μ*m C1 Single-Cell Auto Prep IFC microfluidic chip (Fluidigm), and the chip was then processed on a C1 instrument using the cell-loading script according to the manufacturer’s instructions. Using the standard staining script, the iPSCs were stained with StainAlive TRA-1-60 Antibody (Stemgent, PN 09-0068). The capture efficiency and TRA-1-60 staining were then inspected using the EVOS FL Cell Imaging System (Thermo Fisher) (Supplementary Table S1).

Immediately after imaging, reverse transcription and cDNA amplification were performed in the C1 system using the SMARTer PCR cDNA Synthesis kit (Clontech) and the Advantage 2 PCR kit (Clontech) according to the instructions in the Fluidigm user manual with minor changes to incorporate UMI labeling^20^. Specifically, the reverse transcription primer and the 1:50,000 Ambion^®^ ERCC Spike-In Mix1 (Life Technologies) were added to the lysis buffer, and the template-switching RNA oligos which contain the UMI (5-bp random sequence) were included in the reverse transcription mix^20^,^35^,^36^. When the run finished, full-length, amplified, single-cell cDNA libraries were harvested in a total of approximately 13 *μ*l C1 Harvesting Reagent and quantified using the DNA High Sensitivity LabChip (Caliper). The average yield of samples per C1 plate ranged from 1.26–1.88 ng per microliter (Supplementary Table S1). A bulk sample, a 40 *μ*l aliquot of ~10,000 cells, was collected in parallel with each C1 chip using the same reaction mixes following the C1 protocol (PN 100-7168, Appendix A).

For sequencing library preparation, tagmentation and isolation of 5′ fragments were performed according to the UMI protocol^20^. Instead of using commercially available Tn5 transposase, Tn5 protein stock was freshly purified in house using the IMPACT system (pTXB1, NEB) following the protocol previously described^37^. The activity of Tn5 was tested and shown to be comparable with the EZ-Tn5-Transposase (Epicentre). Importantly, all the libraries in this study were generated using the same batch of Tn5 protein purification. For each of the bulk samples, two libraries were generated using two different indices in order to get sufficient material for sequencing. All 18 bulk libraries were then pooled and labeled as the “bulk” for sequencing.

### Illumina high-throughput sequencing

The scRNA-seq libraries generated from the 96 single cell samples of each C1 chip were pooled and then sequenced in three lanes on an Illumina HiSeq 2500 instrument using the PCR primer (C1-P1-PCR-2: Bio-GAATGATACGGCGACCACCGAT) as the read 1 primer and the Tn5 adapter (C1-Tn5-U: PHO-CTGTCTCTTATACACATCTGACGC) as the index read primer following the UMI protocol^20^.

The master mixes, one mix with all the bulk samples and nine mixes corresponding to the three replicates for the three individuals, were sequenced across four flowcells using a design aimed to minimize the introduction of technical batch effects (Supplementary Table S1). Single-end 100 bp reads were generated along with 8-bp index reads corresponding to the cell-specific barcodes. We did not observe any obvious technical effects due to sequencing lane or flow cell that confounded the inter-individual and inter-replicate comparisons.

### Read mapping

To assess read quality, we ran FastQC (http://www.bioinformatics.babraham.ac.uk/projects/fastqc) and observed a decrease in base quality at the 3′ end of the reads. Thus we removed low quality bases from the 3′ end using sickle with default settings^38^. To handle the UMI sequences at the 5′ end of each read, we used umitools^39^ to find all reads with a UMI of the pattern NNNNNGGG (reads without UMIs were discarded). We then mapped reads to human genome hg19 (only including chromosomes 1–22, X, and Y, plus the ERCC sequences) with Subjunc^40^, discarding non-uniquely mapped reads (option -u). To obtain gene-level counts, we assigned reads to protein-coding genes (Ensembl GRCh37 release 82) and the ERCC spike-in genes using featureCounts^41^. Because the UMI protocol maintains strand information, we required that reads map to a gene in the correct orientation (featureCounts flag -s 1).

In addition to read counts, we utilized the UMI information to obtain molecule counts for the single cell samples. We did not count molecules for the bulk samples because this would violate the assumptions of the UMI protocol, as bulk samples contain far too many unique molecules for the 1,024 UMIs to properly tag them all. First, we combined all reads for a given single cell using samtools^42^. Next, we converted read counts to molecule counts using UMI-tools^43^. UMI-tools counts the number of UMIs at each read start position. Furthermore, it accounts for sequencing errors in the UMIs introduced during the PCR amplification or sequencing steps using a “directional adjacency” method. Briefly, all UMIs at a given read start position are connected in a network using an edit distance of one base pair. However, edges between nodes (the UMIs) are only formed if the nodes have less than a 2x difference in reads. The node with the highest number of reads is counted as a unique molecule, and then it and all connected nodes are removed from the network. This is repeated until all nodes have been counted or removed.

### Filtering cells and genes

We performed multiple quality control analyses to detect and remove data from low quality cells. In an initial analysis investigating the percentage of reads mapping to the ERCC spike-in controls, we observed that replicate 2 of individual NA19098 was a clear outlier (Supplementary Fig. S1). It appeared that too much ERCC spike-in mix was added to this batch, which violated the assumption that the same amount of ERCC molecules was added to each cell. Thus, we removed this batch from all of our analyses.

Next, we kept data from high quality single cells that passed the following criteria:

- Only one cell observed per well.
- At least 1,556,255 mapped reads.
- Less than 36.4% unmapped reads.
- Less than 3.2% ERCC reads.
- More than 6,788 genes with at least one read.

We chose the above criteria based on the distribution of these metrics in the empty wells (the cutoff is the 95th percentile, Supplementary Fig. S1). In addition, we observed that some wells classified as containing only one cell were clustered with multi-cell wells when plotting 1) the number of gene molecules versus the concentration of the samples, and 2) the read to molecule conversion efficiency (total molecule number divided by total read number) of endogenous genes versus that of ERCC. We therefore established filtering criteria for these misidentified single-cell wells using linear discriminant analysis (LDA). Specifically, LDA was performed to classify wells into empty, one-cell, and two-cell using the discriminant functions of 1) sample concentration and the number of gene molecules, and 2) endogenous and ERCC gene read to molecule conversion efficiency (Supplementary Fig. S2). After filtering, we maintained 564 high quality single cells (NA19098: 142, NA19101: 201, NA19239: 221).

The quality control analyses were performed using all protein-coding genes (Ensembl GRCh37 release 82) with at least one observed read. Using the high quality single cells, we further removed genes with low expression levels for downstream analyses. We removed all genes with a mean log_2_ cpm less than 2, which did not affect the relative differences in the proportion of genes detected across batches (Supplementary Fig. S10). We also removed genes with molecule counts larger than 1,024 for the correction of collision probability. In the end we kept 13,058 endogenous genes and 48 ERCC spike-in genes.

### Calculate the input molecule quantities of ERCC spiked-ins

According to the information provided by Fluidigm, each of the 96 capture chamber received 13.5 nl of lysis buffer, which contain 1:50,000 Ambion^®^ ERCC Spike-In Mix1 (Life Technologies) in our setup. Therefore, our estimation of the total spiked-in molecule number was 16,831 per sample. Since the relative concentrations of the ERCC genes were provided by the manufacturer, we were able to calculate the molecule number of each ERCC gene added to each sample. We observed that the levels of ERCC spike-ins strongly correlated with the input quantities (r = 0.9914, Fig. 1g). The capture efficiency, defined as the fraction of total input molecules being successfully detected in each high quality cell, had an average of 6.1%.

### Subsampling

We simulated different sequencing depths by randomly subsampling reads and processing the subsampled data through the same pipeline described above to obtain the number of molecules per gene for each single cell. To assess the impact of sequencing depth and number of single cells, we calculated the following three statistics:

1. The Pearson correlation of the gene expression level estimates from the single cells compared to the bulk samples. For the single cells, we summed the gene counts across all the samples and then calculated the log_2_ cpm of this pseudo-bulk. For the bulk samples, we calculated the log_2_ cpm separately for each of the three replicates and then calculated the mean per gene.
2. The number of genes detected with at least one molecule in at least one cell.
3. The Pearson correlation of the cell-to-cell gene expression variance estimates from the subsampled single cells compared to the variance estimates using the full single cell data set.

Each data point in Fig. 2 represents the mean +/− the standard error of the mean (SEM) of 10 random subsamples of cells. We split the genes by expression level into two groups (6,097 genes each) to highlight that most of the improvement with increased sequencing depth and number of cells was driven by the estimates of the lower half of expressed genes. The data shown is for individual NA19239, but the results were consistent for individuals NA19098 and NA19101. Only high quality single cells (Supplementary Table S2) were included in this analysis.

### A framework for testing individual and batch effects

Individual effect and batch effect between the single cell samples were evaluated in a series of analyses that examine the potential sources of technical variation on gene expression measurements. These analyses took into consideration that in our study design, sources of variation between single cell samples naturally fall into a hierarchy of individuals and C1 batches. In these sample-level analyses, the variation introduced at both the individual-level and the batch-level was modeled in a nested framework that allows random noise between C1 batches within individuals. Specifically, for each cell sample in individual *i*, replicate *j* and well *k*, we used *y*_*ijk*_ to denote some sample measurement (e.g. total molecule-counts) and fit a linear mixed model with the fixed effect of individual *α*_*i*_ and the random effect of batch *b*_*ij*_:


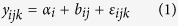


where the random effect *b*_*ij*_ of batch follows a normal distribution with mean zero and variance 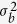, and *ε*_*ijk*_ describes residual variation in the sample measurement. To test the statistical significance of individual effect (i.e., null hypothesis *α*_1_ = *α*_2_ = *α*_3_), we performed a likelihood ratio test (LRT) to compare the above full model and the reduced model that excludes *α*_1_. To test if there was a batch effect (i.e., null hypothesis 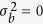), we performed an F-test to compare the variance that is explained by the above full model and the variance due to the reduced model that excludes *b*_*ij*_.

The nested framework was applied to test the individual and batch effects between samples in the following cases. The data includes samples after quality control and filtering.

1. Total molecule count (on the log_2_ scale) was modeled as a function of individual effect and batch effect, separately for the ERCC spike-ins and for the endogenous genes.
2. Read-to-molecule conversion efficiency was modeled as a function of individual effect and batch effect, separately for the ERCC spike-ins and for the endogenous genes.

### Estimating variance components for per-gene expression levels

To assess the relative contributions of individual and technical variation, we analyzed per-gene expression profiles and computed variance component estimates for the effects of individual and C1 batch (Supplementary Fig. S4). The goal here was to quantify the proportion of cell-to-cell variance due to individual (biological) effect and to C1 batch (technical) at the per-gene level. Note that the goal here was different from that of the previous section, where we simply tested for the existence of individual and batch effects at the sample level by rejecting the null hypothesis of no such effects. In contrast, here we fit a linear mixed model per gene where the dependent variable was the gene expression level (log_2_ counts per million) and the independent variables were individual and batch, both modeled as random effects.

The variance parameters of individual effect and batch effect were estimated using a maximum penalized likelihood approach^44^, which can effectively avoid the common issue of zero variance estimates due to small sample sizes (there were three individuals and eight batches). We used the blmer function in the R package blme and set the penalty function to be the logarithm of a gamma density with shape parameter = 2 and rate parameter tending to zero.

The estimated variance components were used to compute the sum of squared deviations for individual and batch effects. The proportion of variance due to each effect is equal to the relative contribution of the sum of squared deviations for each effect compared to the total sum of squared deviations per gene. Finally, we compared the estimated proportions of variance due to the individual effect and the batch effect, across genes, using a non-parametric one-way analysis of variance (Kruskal-Wallis rank sum test).

### Normalization

We transformed the single cell molecule counts in multiple steps (Fig. 4). First, we corrected for the collision probability using a method similar to that developed by Grün *et al*.^12^. Essentially we corrected for the fact that we did not observe all the molecules originally in the cell. The main difference between our approach and that of Grün *et al*.^12^ was that we applied the correction at the level of gene counts and not individual molecule counts. Second, we standardized the molecule counts to log_2_ counts per million (cpm). This standardization was performed using only the endogenous gene molecules and not the ERCC molecules. Third, we corrected for cell-to-cell technical noise using the ERCC spike-in controls. For each single cell, we fit a Poisson generalized linear model (GLM) with the log_2_ expected ERCC molecule counts as the independent variable, and the observed ERCC molecule counts as the dependent variable, using the standard log link function. Next we used the slope and intercept of the Poisson GLM regression line to transform the log_2_ cpm for the endogenous genes in that cell. This is analogous to the standard curves used for qPCR measurements, but taking into account that lower concentration ERCC genes will have higher variance from Poisson sampling. Fourth, we removed technical noise between the eight batches (three replicates each for NA19101 and NA19239 and two replicates for NA19098). We fit a linear mixed model with a fixed effect for individual and a random effect for the eight batches and removed the variation captured by the random effect (see the next section for a detailed explanation).

For the bulk samples, we used read counts even though the reads contained UMIs. Because these samples contained RNA molecules from ~10,000 cells, we could not assume that the 1,024 UMIs were sufficient for tagging such a large number of molecules. We standardized the read counts to log_2_ cpm.

### Removal of technical batch effects

Our last normalization step adjusted the transformed log_2_ gene expression levels for cell-to-cell correlation within each C1 plate. The algorithm mimics a method that was initially developed for adjusting within-replicate correlation in microarray data^24^. We assumed that for each gene *g*, cells that belong to the same batch *j* are correlated, for batches *j* = 1, …, 8. We also assume that the cell-to-cell gene expression variation due to C1 batch effect is independent of biological variation between individuals.

We fit a linear mixed model for each gene *g* that includes a fixed effect of individual and a random effect for within-batch variation attributed to cell-to-cell correlation in each C1 plate:


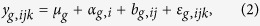


where *y*_*g*,*ijk*_ denotes log_2_ counts-per-million (cpm) of gene *g* in individual *i*, replicate *j*, and cell *k; i* = *NA*19098, *NA*19101, *NA*19239, *j* = 1, …, *n*_*i*_ with *n*_*i*_ the number of replicates in individual *i, k* = 1, …, *n*_*ij*_ with *n*_*ij*_ the number of cells in individual *i* replicate *j. μ*_*g*_ denotes the mean gene expression level across cells, *α*_*g*,*i*_ quantifies the individual effect on mean gene expression, *b*_*g*,*ij*_ models the replicate effect on mean expression level (assumed to be stochastic, independent, and identically distributed with mean 0 and variance 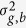). Finally, *ε*_*g*,*ijk*_ describes the residual variation in gene expression.

Batch-corrected expression levels were computed as


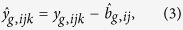


where 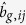 are the least-squares estimates. The computations in this step were done with the gls.series function of the limma package^45^. We note that the batch correction method described here relies on the assumption that the technical effect due to batch is identical across individuals (no interaction). This assumption may be violated, yet due to the small number of individuals in our study, we are unable to explicitly test for a first order interaction between batch and individual. In the future, the issue of confounding effect between individual and batch may be more effectively addressed in the design of the study, instead of increasing the sample size. Indeed, in the main text we describe a study design that can effectively address the issue of confounding effect by processing samples from multiple individuals in a single C1 plate.

### Measurement of gene expression noise

While examining gene expression noise (using the coefficient of variation or CV) as a function of mean RNA abundance across C1 replicates, we found that the CV of molecule counts among endogenous genes and ERCC spike-in controls suggested similar expression variability patterns. Both endogenous and ERCC spike-in control CV patterns approximately followed an over-dispersed Poisson distribution (Supplementary Fig. S11), which is consistent with previous studies^11^,^20^. We computed a measure of gene expression noise that is independent of RNA abundance across individuals^46^,^47^. First, squared coefficients of variation (CVs) for each gene were computed for each individual and also across individuals, using the batch-corrected molecule data. Then we computed the distance of individual-specific CVs to the rolling median of global CVs among genes that have similar RNA abundance levels. These transformed individual CV values were used as our measure of gene expression noise. Specifically, we computed the adjusted CV values as follows:

1. Compute squared CVs of molecule counts in each individual and across individuals.
2. Order genes by the global average molecule counts.
3. Starting from the genes with the lowest global average gene expression level, for every sliding window of 50 genes, subtract log_10_ median squared CVs from log_10_ squared CVs of each cell line, and set 25 overlapping genes between windows. The computation was performed with the rollapply function of the R zoo package^48^. After this transformation step, CV no longer had a polynomial relationship with mean gene molecule count (Supplementary Fig. S11).

### Identification of genes associated with inter-individual differences in regulatory noise

To identify differential noise genes across individuals, we computed median absolute deviation (MAD) - a robust and distribution-free dissimilarity measure for gene *g*:


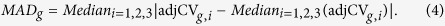


Large values of *MAD*_*g*_ suggest a large deviation from the median of the adjusted CV values. We identified genes with significant inter-individual differences using a permutation-based approach. Specifically, for each gene, we computed empirical *P*-values based on 300,000 permutations. In each permutation, the sample of origin labels were shuffled between cells. Because the number of permutations in our analysis was smaller than the maximum possible number of permutations, we computed the empirical *P*-values as 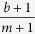, where *b* is the number of permuted MAD values greater than the observed MAD value, and *m* is the number of permutations. Adding 1 to *b* avoided an empirical *P*-value of zero^49^.

### Gene enrichment analysis

We used ConsensusPATHDB^50^ to identify GO terms that are over-represented for genes whose variation in single cell expression levels were significantly difference between individuals.

### Individual assignment based on scRNA-seq reads

We were able to successfully determine the correct identity of each single cell sample by examining the SNPs present in their RNA sequencing reads. Specifically, we used the method verifyBamID (https://github.com/statgen/verifyBamID) developed by Jun *et al*.^27^, which detects sample contamination and/or mislabeling by comparing the polymorphisms observed in the sequencing reads for a sample to the genotypes of all individuals in a study. For our test, we included the genotypes for all 120 Yoruba individuals that are included in the International HapMap Project^28^. The genotypes included the HapMap SNPs with the 1000 Genomes Project SNPs^51^ imputed, as previously described^52^. We subset to include only the 528,289 SNPs that overlap Ensembl protein-coding genes. verifyBamID used only 311,848 SNPs which passed its default thresholds (greater than 1% minor allele frequency and greater than 50% call rate). Using the option –best to return the best matching individual, we obtained 100% accuracy identifying the single cells of all three individuals (Supplementary Fig. S9).

### Data and code availability

The data have been deposited in NCBI’s Gene Expression Omnibus^53^ and are accessible through GEO Series accession number GSE77288 (http://www.ncbi.nlm.nih.gov/geo/query/acc.cgi?acc=GSE77288). The code and processed data are available at https://github.com/jdblischak/singleCellSeq. The results of our analyses are viewable at https://jdblischak.github.io/singleCellSeq/analysis.

## Additional Information

**How to cite this article**: Tung, P.-Y. *et al*. Batch effects and the effective design of single-cell gene expression studies. *Sci. Rep.* **7**, 39921; doi: 10.1038/srep39921 (2017).

**Publisher's note:** Springer Nature remains neutral with regard to jurisdictional claims in published maps and institutional affiliations.

## Supplementary Material

Supplementary Information

Supplementary Table S2

Supplementary Table S3

Supplementary Table S4

## References

[b1] Macaulay I. C. & Voet T. . Single cell genomics: advances and future perspectives. PLoS Genet 10, e1004126 (2014). 24497842 10.1371/journal.pgen.1004126 PMC3907301

[b2] Saliba A. E., Westermann A. J., Gorski S. A. & Vogel J.. Single-cell RNA-seq: advances and future challenges. Nucleic Acids Res 42, 8845–60 (2014). 25053837 10.1093/nar/gku555 PMC4132710

[b3] Macosko E. Z. . Highly parallel genome-wide expression profiling of individual cells using nanoliter droplets. Cell 161, 1202–14 (2015). 26000488 10.1016/j.cell.2015.05.002 PMC4481139

[b4] Handel A. E. . Assessing similarity to primary tissue and cortical layer identity in induced pluripotent stem cell-derived cortical neurons through single-cell transcriptomics. Hum Mol Genet 25, 989–1000 (2016). 26740550 10.1093/hmg/ddv637 PMC4754051

[b5] Drissen R. . Distinct myeloid progenitor-differentiation pathways identified through single-cell RNA sequencing. Nat Immunol doi: 10.1038/ni.3412 (2016). PMC4972405 27043410

[b6] Shalek A. K. . Single-cell transcriptomics reveals bimodality in expression and splicing in immune cells. Nature 498, 236–40 (2013). 23685454 10.1038/nature12172 PMC3683364

[b7] Jaitin D. A. . Massively parallel single-cell RNA-seq for marker-free decomposition of tissues into cell types. Science 343, 776–9 (2014). 24531970 10.1126/science.1247651 PMC4412462

[b8] Miyamoto D. T. . RNA-seq of single prostate cTCs implicates noncanonical wnt signaling in antiandrogen resistance. Science 349, 1351–6 (2015). 26383955 10.1126/science.aab0917 PMC4872391

[b9] Kim K. T. . Single-cell mRNA sequencing identifies subclonal heterogeneity in anti-cancer drug responses of lung adenocarcinoma cells. Genome Biol 16, 127 (2015). 26084335 10.1186/s13059-015-0692-3 PMC4506401

[b10] Stegle O., Teichmann S. A. & Marioni J. C. . Computational and analytical challenges in single-cell transcriptomics. Nat Rev Genet 16, 133–45 (2015). 25628217 10.1038/nrg3833

[b11] Brennecke P. . Accounting for technical noise in single-cell RNA-seq experiments. Nat Methods 10, 1093–5 (2013). 24056876 10.1038/nmeth.2645

[b12] Grün D., Kester L. & Oudenaarden A. van . Validation of noise models for single-cell transcriptomics. Nat Methods 11, 637–40 (2014). 24747814 10.1038/nmeth.2930

[b13] Jiang L. . Synthetic spike-in standards for RNA-seq experiments. Genome Res 21, 1543–51 (2011). 21816910 10.1101/gr.121095.111 PMC3166838

[b14] Ding B. . Normalization and noise reduction for single cell RNA-seq experiments. Bioinformatics 31, 2225–7 (2015). 25717193 10.1093/bioinformatics/btv122 PMC4481848

[b15] Vallejos C. A., Marioni J. C. & Richardson S. . BASiCS: Bayesian analysis of single-cell sequencing data. PLoS Comput Biol 11, e1004333 (2015). 26107944 10.1371/journal.pcbi.1004333 PMC4480965

[b16] Kivioja T. . Counting absolute numbers of molecules using unique molecular identifiers. Nat Methods 9, 72–4 (2012). 10.1038/nmeth.1778 22101854

[b17] Fu G. K., Hu J., Wang P. H. & Fodor S. P. . Counting individual dNA molecules by the stochastic attachment of diverse labels. Proc Natl Acad Sci USA 108, 9026–31 (2011). 21562209 10.1073/pnas.1017621108 PMC3107322

[b18] Casbon J. A., Osborne R. J., Brenner S. & Lichtenstein C. P. . A method for counting PCR template molecules with application to next-generation sequencing. Nucleic Acids Res 39, e81 (2011). 21490082 10.1093/nar/gkr217 PMC3130290

[b19] Shiroguchi K., Jia T. Z., Sims P. A. & Xie X. S. . Digital RNA sequencing minimizes sequence-dependent bias and amplification noise with optimized single-molecule barcodes. Proc Natl Acad Sci USA 109, 1347–52 (2012). 22232676 10.1073/pnas.1118018109 PMC3268301

[b20] Islam S. . Quantitative single-cell RNA-seq with unique molecular identifiers. Nature methods 11, 163–6 (2014). 24363023 10.1038/nmeth.2772

[b21] Wu A. R. . Quantitative assessment of single-cell RNA-sequencing methods. Nat Methods 11, 41–6 (2014). 24141493 10.1038/nmeth.2694 PMC4022966

[b22] Klein A. M. . Droplet barcoding for single-cell transcriptomics applied to embryonic stem cells. Cell 161, 1187–201 (2015). 26000487 10.1016/j.cell.2015.04.044 PMC4441768

[b23] Hicks S. C., Teng M. & Irizarry R. A. . On the widespread and critical impact of systematic bias and batch effects in single-cell RNA-seq data. bioRxiv doi: 10.1101/025528 (2015).

[b24] Smyth, M., G. K. & Scott H. S. . User of within-array replicate spots for assessing differential expression in microarray experiments. Bioinformatics 21, 2067–75 (2005). 15657102 10.1093/bioinformatics/bti270

[b25] Raser J. M. & O’Shea E. K. . Noise in gene expression: origins, consequences, and control. Science 309, 2010–3 (2005). 16179466 10.1126/science.1105891 PMC1360161

[b26] Fehrmann S. . Natural sequence variants of yeast environmental sensors confer cell-to-cell expression variability. Mol Syst Biol 9, 695 (2013). 24104478 10.1038/msb.2013.53 PMC3817403

[b27] Jun G. . Detecting and estimating contamination of human dNA samples in sequencing and array-based genotype data. American journal of human genetics 91, 839–48 (2012). 23103226 10.1016/j.ajhg.2012.09.004 PMC3487130

[b28] The International Hapmap Consortium . A haplotype map of the human genome. Nature 437, 1299–320 (2005). 16255080 10.1038/nature04226 PMC1880871

[b29] Pollen A. A. . Low-coverage single-cell mRNA sequencing reveals cellular heterogeneity and activated signaling pathways in developing cerebral cortex. Nat Biotechnol 32, 1053–8 (2014). 25086649 10.1038/nbt.2967 PMC4191988

[b30] Risso D., Ngai J., Speed T. P. & Dudoit S. . Normalization of RNA-seq data using factor analysis of control genes or samples. Nat Biotechnol 32, 896–902 (2014). 25150836 10.1038/nbt.2931 PMC4404308

[b31] SEQC/MAQC-III Consortium . A comprehensive assessment of RNA-seq accuracy, reproducibility and information content by the sequencing quality control consortium. Nature biotechnology 32, 903–14 (2014). 10.1038/nbt.2957 PMC4321899 25150838

[b32] Borel C. . Biased allelic expression in human primary fibroblast single cells. American Journal of Human Genetics 96, 70–80 (2015). 25557783 10.1016/j.ajhg.2014.12.001 PMC4289680

[b33] Finak G. . MAST: a flexible statistical framework for assessing transcriptional changes and characterizing heterogeneity in single-cell rNA sequencing data. Genome Biology 16, 1–13 (2015). 26653891 10.1186/s13059-015-0844-5 PMC4676162

[b34] Chen G. . Chemically defined conditions for human iPSC derivation and culture. Nature methods 8, 424–9 (2011). 21478862 10.1038/nmeth.1593 PMC3084903

[b35] Islam S. . Characterization of the single-cell transcriptional landscape by highly multiplex RNA-seq. Genome Res 21, 1160–7 (2011). 21543516 10.1101/gr.110882.110 PMC3129258

[b36] Islam S. . Highly multiplexed and strand-specific single-cell RNA 5′ end sequencing. Nat Protoc 7, 813–28 (2012). 22481528 10.1038/nprot.2012.022

[b37] Picelli S. . Tn5 transposase and tagmentation procedures for massively scaled sequencing projects. Genome research 24, 2033–40 (2014). 25079858 10.1101/gr.177881.114 PMC4248319

[b38] Joshi N. & Fass J. . Sickle: A sliding-window, adaptive, quality-based trimming tool for fastQ files (version 1.33) [software]. *Available at* https://github.com/najoshi/sickle (2011).

[b39] Brown J., Hesselberth J. & Blischak J. . umitools v2.1.1., doi: 10.5281/zenodo.34933 (2015).

[b40] Liao Y., Smyth G. K. & Shi W. . The subread aligner: fast, accurate and scalable read mapping by seed-and-vote. Nucleic acids research 41, e108 (2013). 23558742 10.1093/nar/gkt214 PMC3664803

[b41] Liao Y., Smyth G. K. & Shi W. . featureCounts: an efficient general purpose program for assigning sequence reads to genomic features. Bioinformatics (Oxford, England ) 30, 923–30 (2014). 10.1093/bioinformatics/btt656 24227677

[b42] Li H. . The sequence alignment/map format and sAMtools. Bioinformatics (Oxford, England ) 25, 2078–9 (2009). 10.1093/bioinformatics/btp352 PMC2723002 19505943

[b43] Smith T. S., Heger A. & Sudbery I. . UMI-tools: Modelling sequencing errors in Unique Molecular Identifiers to improve quantification. bioRxiv, doi: http://dx.doi.org/10.1101/051755(2016). 10.1101/gr.209601.116 PMC5340976 28100584

[b44] Chung Y., Rabe-Hesketh S., Dorie V., Gelman A. & Liu J. . A non-degenerate estimator for hierarchical variance parameters via penalized likelihood estimation. Psychometrika 78, 685–709 (2013). 24092484 10.1007/s11336-013-9328-2

[b45] Ritchie, P., M. E. & Smyth G. K. . limma powers differential expression analysis for RNA-sequencing and microarray studies. Nucleic Acids Research 43, e47 (2015). 25605792 10.1093/nar/gkv007 PMC4402510

[b46] Kolodziejczyk, K., A. A. & Marioni J. C. . Single cell RNA-sequencing of pluripotent states unlocks modular transcriptional variation. Cell Stem Cell 17, 471–485 (2015). 26431182 10.1016/j.stem.2015.09.011 PMC4595712

[b47] Newman, G., J. R. S. & Weissman J. S. . Single-cell proteomic analysis of s. cerevisiae reveals the architecture of biological noise. Nature 441, 840–846 (2006). 16699522 10.1038/nature04785

[b48] Zeileis A. & Grothendieck G. . zoo: S3 infrastructure for regular and irregular time series. Journal of Statistical Software 14, 1–27 (2005).

[b49] Phipson B. & Smyth G. K. . Permutation p-values should never be zero: calculating exact p-values when permutations are randomly drawn. Statistical applications in genetics and molecular biology 9, Article 39 (2010). 10.2202/1544-6115.1585 21044043

[b50] Kamburov P. A. . ConsensusPathDB: toward a more complete picture of cell biology. Nucleic Acids Research 39, D712–717 (2011). 21071422 10.1093/nar/gkq1156 PMC3013724

[b51] The 1000 Genomes Project Consortium . An integrated map of genetic variation from 1,092 human genomes. Nature 491, 56–65 (2012). 23128226 10.1038/nature11632 PMC3498066

[b52] McVicker G. . Identification of genetic variants that affect histone modifications in human cells. Science (New York, N.Y.) 342, 747–9 (2013). 10.1126/science.1242429 PMC3947669 24136359

[b53] Edgar R., Domrachev M. & Lash A. E. . Gene expression omnibus: NCBI gene expression and hybridization array data repository. Nucleic acids research 30, 207–210 (2002). 11752295 10.1093/nar/30.1.207 PMC99122

