# Supplementary material for: Batch effects and the effective design of single-cell gene expression studies
Source: Sci Rep. 2017 Jan 3;7:39921. doi: 10.1038/srep39921 (PMC5206706; doi:10.1038/srep39921)
Supplement: Supplementary Information [file srep39921-s1.pdf]

Supplementary Information

Batch effects and the effective design of single-cell gene expression studies

Po-Yuan Tung[1,8], John D. Blischak[1,2,8], Chiaowen Joyce Hsiao[1,8], David A. Knowles[3,4], Jonathan E. Burnett[1], Jonathan K. Pritchard[3,5,6], Yoav Gilad[1,7]*

[1]Department of Human Genetics, University of Chicago, Chicago, Illinois, USA
[2]Committee on Genetics, Genomics, and Systems Biology, University of Chicago, Chicago, Illinois, USA
[3]Department of Genetics, Stanford University, Stanford, CA, USA
[4]Department of Radiology, Stanford University, Stanford, CA, USA
[5]Department of Biology, Stanford University, Stanford, CA, USA
[6]Howard Hughes Medical Institute, Stanford University, CA, USA
[7]Department of Medicine, University of Chicago, Chicago, Illinois, USA
[8]These authors contributed equally to this work

*Correspondence should be addressed to YG (gilad@uchicago.edu).

Supplementary Figures

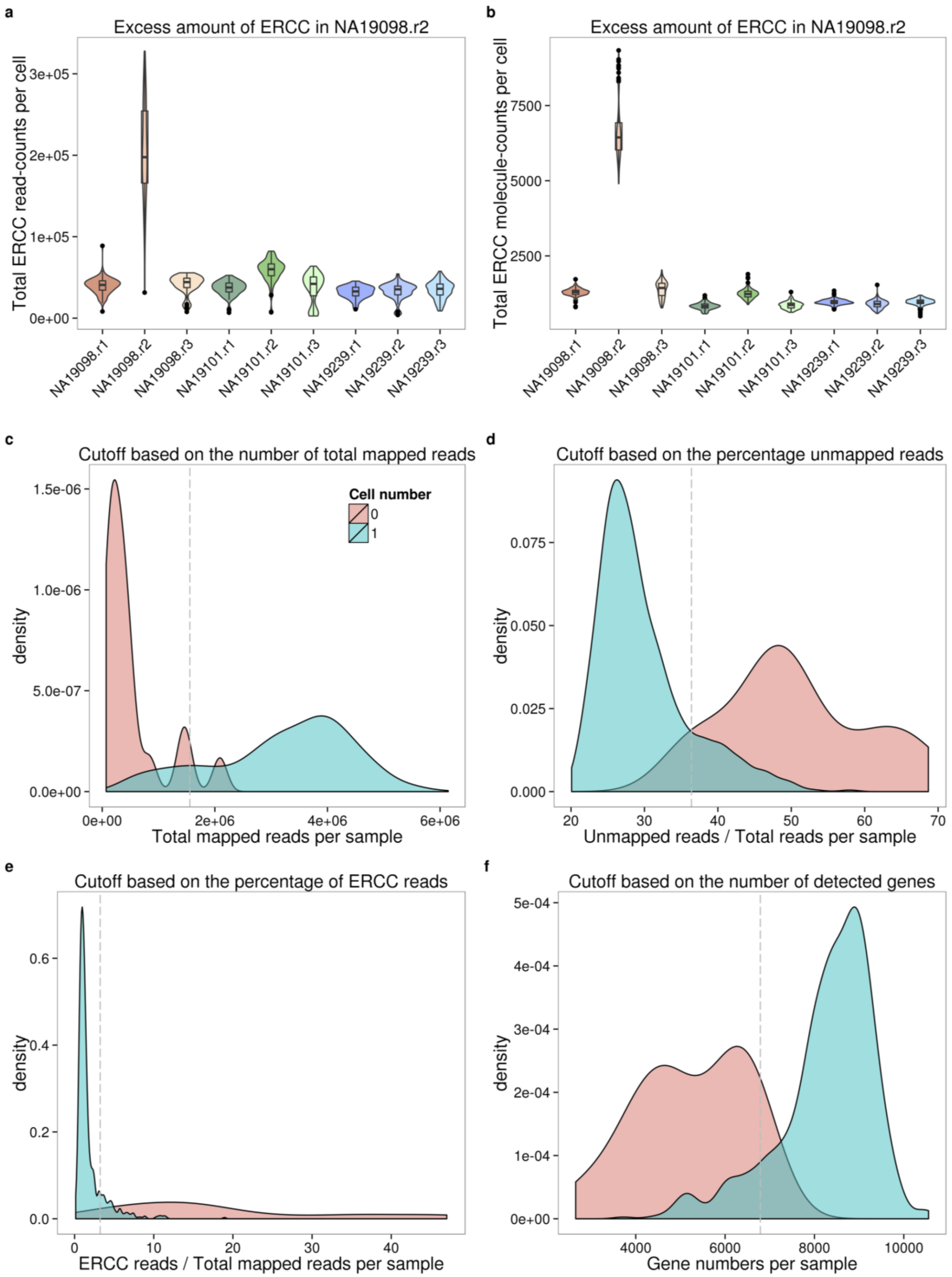

Supplementary Figure S1. Removal of low quality samples. Violin plots of the total read-counts of ERCC spike-in controls in (A) and the total molecule-counts in (B) in single cell samples. The three colors represent the three individuals (NA19098 in red, NA19101 in green, and NA19239 in blue). (C-F) Density plots of the distributions of the total mapped reads in (C),

the percentage of unmapped reads in (D), the percentage of ERCC reads in (E), and the number of detected genes in (F). The dash lines indicate the cutoffs based on the 95th percentile of the samples with no cells.

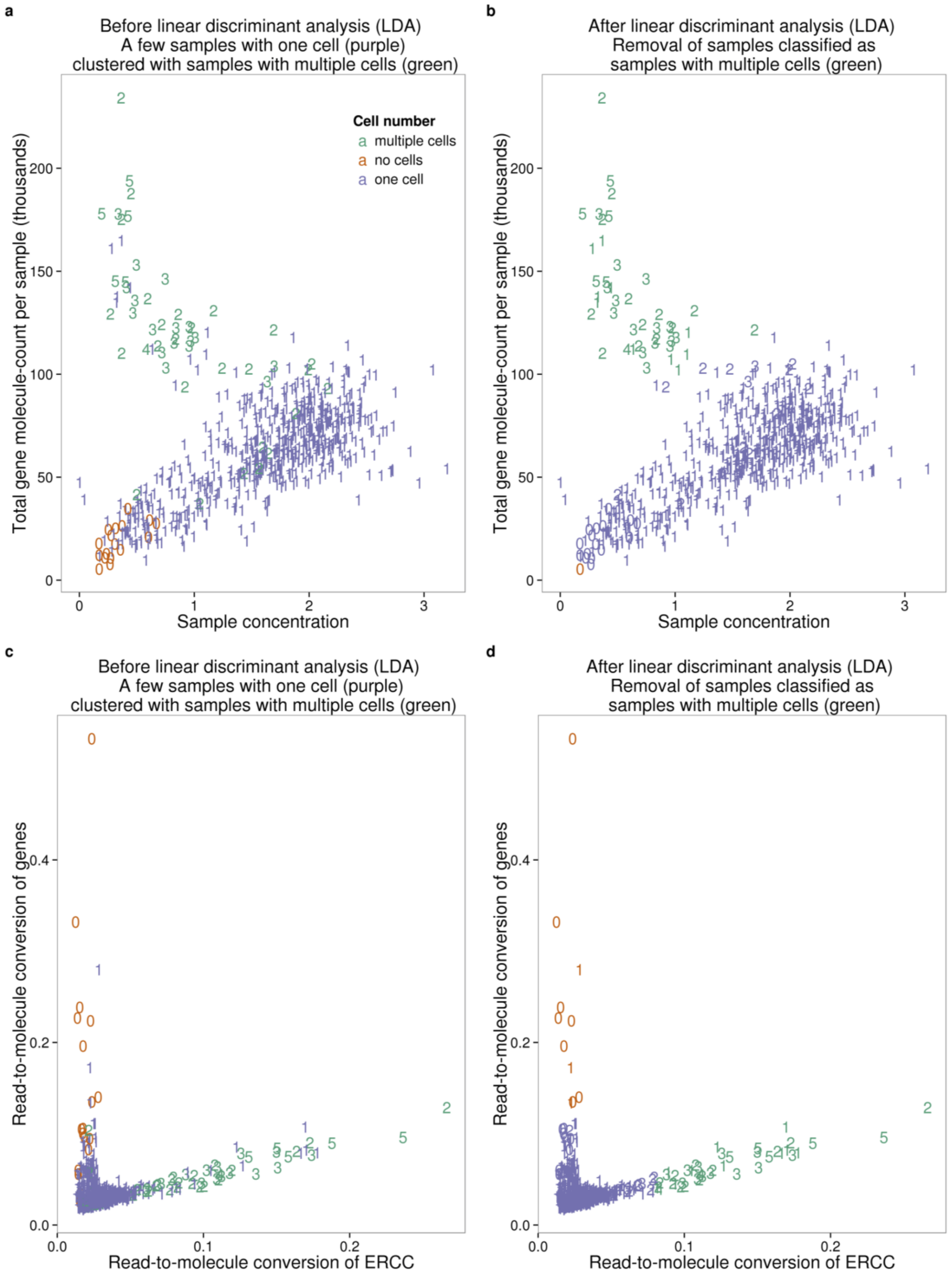

Supplementary Figure S2. Removal of samples with multiple cells. Scatterplots of the three groups of samples (no cell in green, single-cell in orange, and two or more cells in purple)

before (A) and after (B) the linear discriminant analysis (LDA) using sample concentration of cDNA amplicons (ng/ l) and the number of total gene molecule-counts. (C and D) Similarly, LDA was performed to identify potential multi-cell samples using the read-to-molecule conversion efficiency (total molecule-counts divided by total read-counts per sample) of endogenous genes and ERCC spike-in controls. Scatterplots of before and after the LDA in (C) and (D), respectively. The numbers indicate the number of cells observed in each cell capture site.

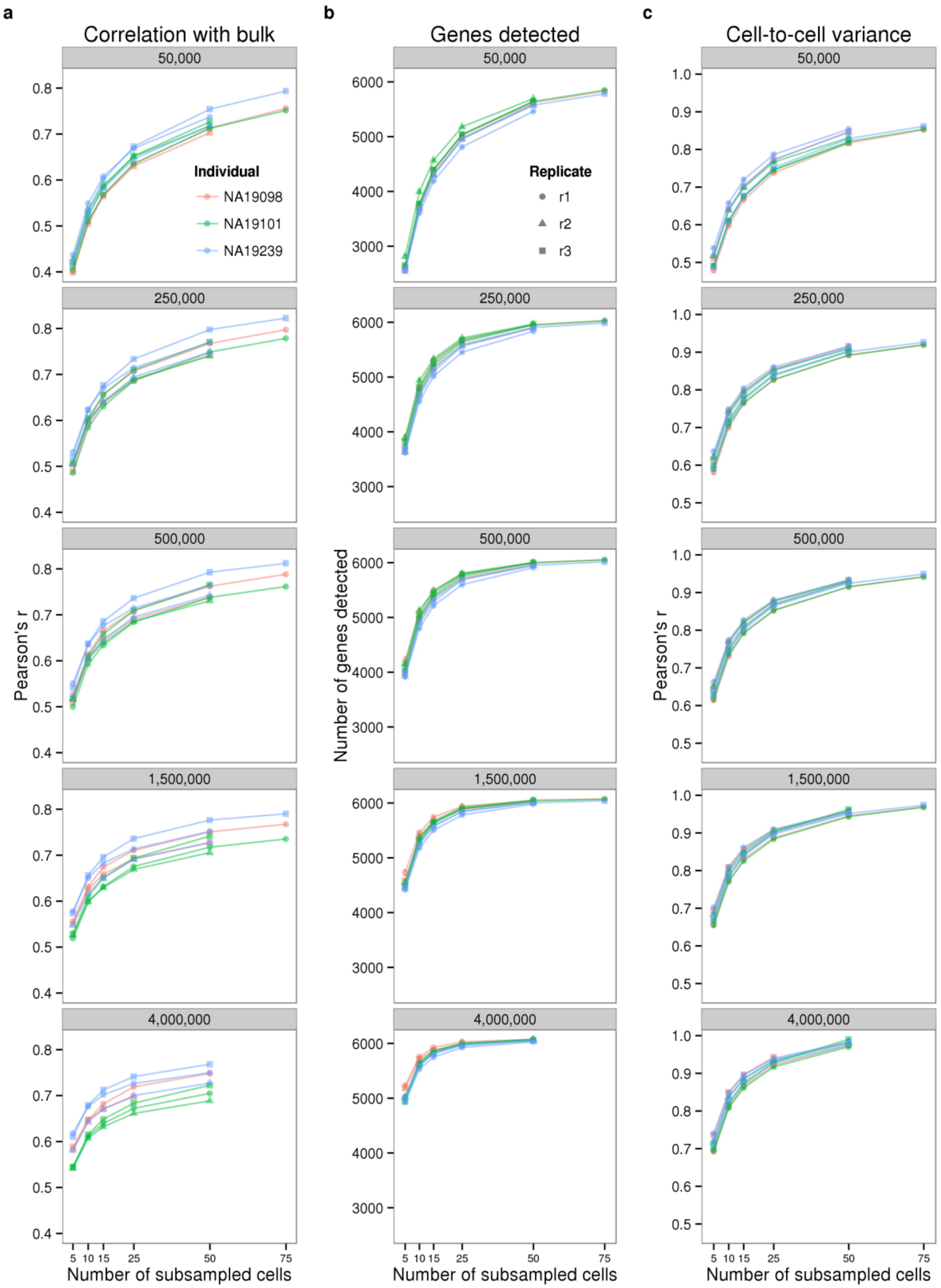

Supplementary Figure S3. The effect of sequencing depth and cell number on single cell UMI estimates is consistent across batches. The sequencing reads from each C1 batch were subsampled to the indicated sequencing depth and cell number, and subsequently converted to

molecules using the UMIs. The x-axis is the number of subsampled cells, the y-axis is the metric measured for each subsample, and each line corresponds to one C1 batch. Each point represents the mean of 10 random draws of the indicated cell number (the SEM was omitted because it was too small to be easily visible). The colors represent the three individuals (NA19098 is in red, NA19101 in green, and NA19239 in blue). Data from different C1 replicates is plotted in different shapes. Only high quality cells were included (thus there is no r2 for NA19098). The number at the top of each subplot indicates the number of subsampled sequencing reads, which increases from top to bottom. Each subpanel shows the results for quantifying the 6,097 (50% of detected) genes with lower expression levels, since there was minimal variation from different subsamples for the top 50% of genes with higher expression levels. (A) Pearson correlation of aggregated gene expression level estimates from single cells from one C1 chip compared to the mathced bulk sequencing sample. (B) Total number of genes detected with at least one molecule in at least one of the single cells. (C) Pearson correlation of cell-to-cell gene expression variance estimates from subsets of single cells compared to all single cells from the C1 chip.

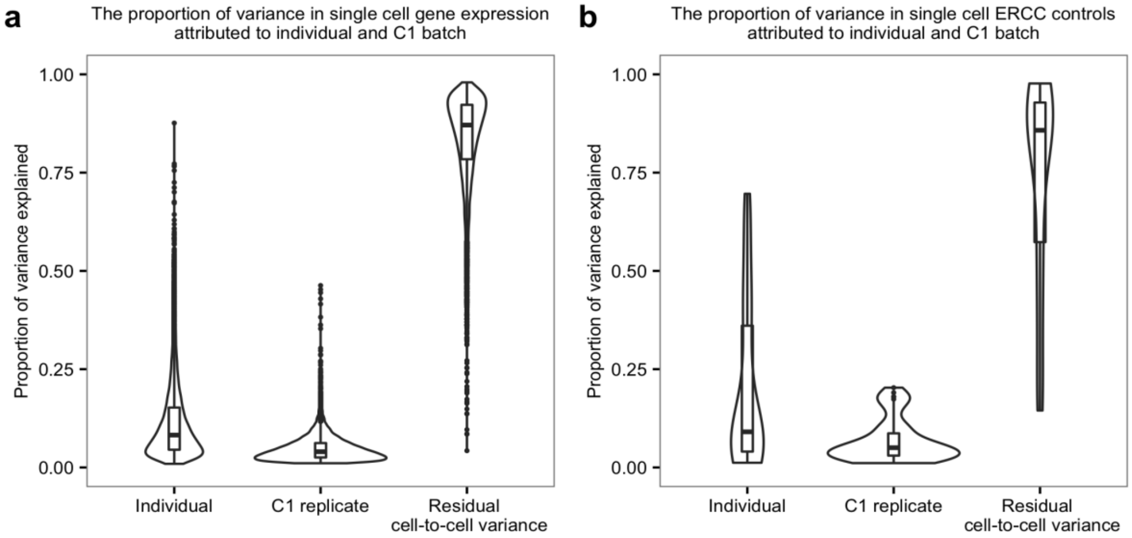

Supplementary Figure S4. Sources of cell-to-cell variance in per-gene expression profile. Violin plots of the proportion of per-gene cell-to-cell variance that was due to individual sample of origin, different C1 replicates, and other single cell sample differences. These results were calculated from the molecule counts before normalization and batch correction. Endogenous genes are shown in (A) and the ERCC spike-in controls in (B).

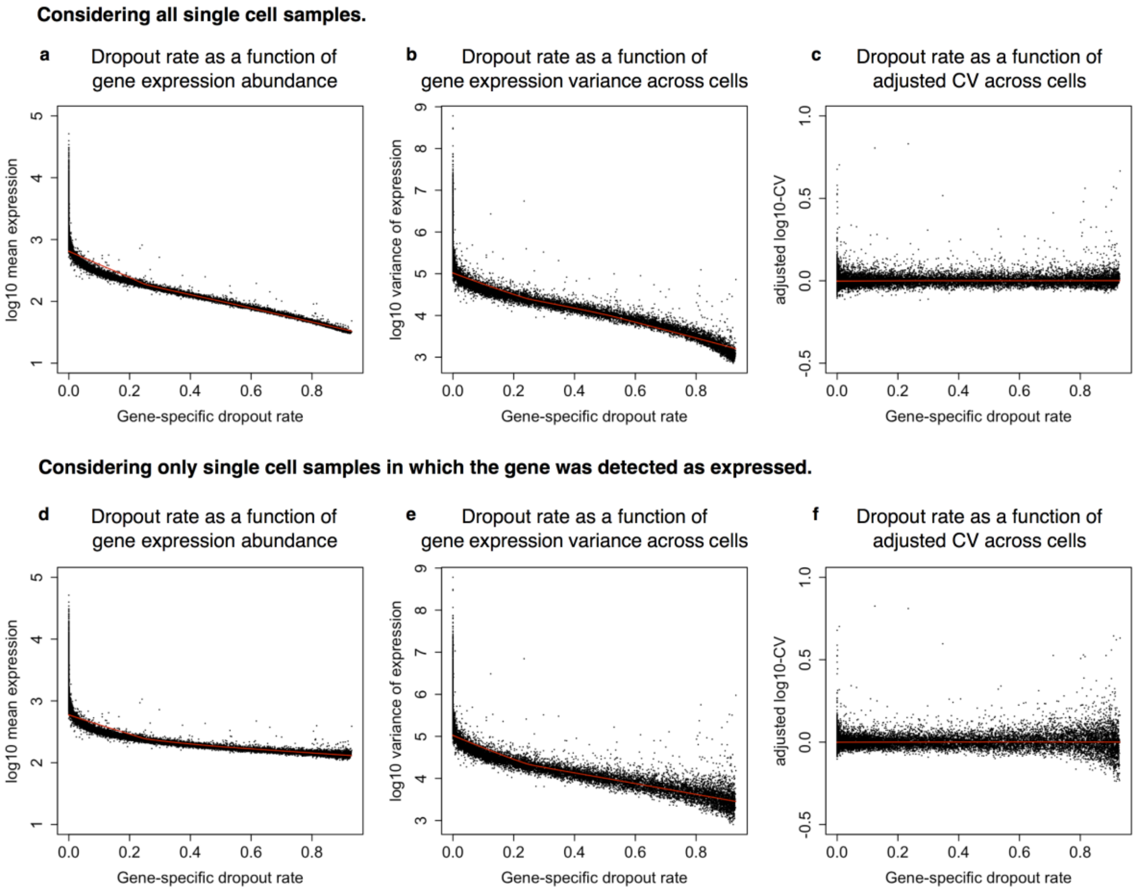

Supplementary Figure S5. The gene-specific dropout rate. The gene-specific dropout rate (the proportion of cells in which the gene is undetected) and its relationship with $\log_{10}$ mean expression, with $\log_{10}$ variance of expression, and with the adjusted log10-CV when the analysis includes (A-C) and excludes (D-F) samples in which the gene was not detected as expressed. Each point represents a gene, and red lines indicate the predicted values using locally weighted scatterplot smoothing (LOESS).

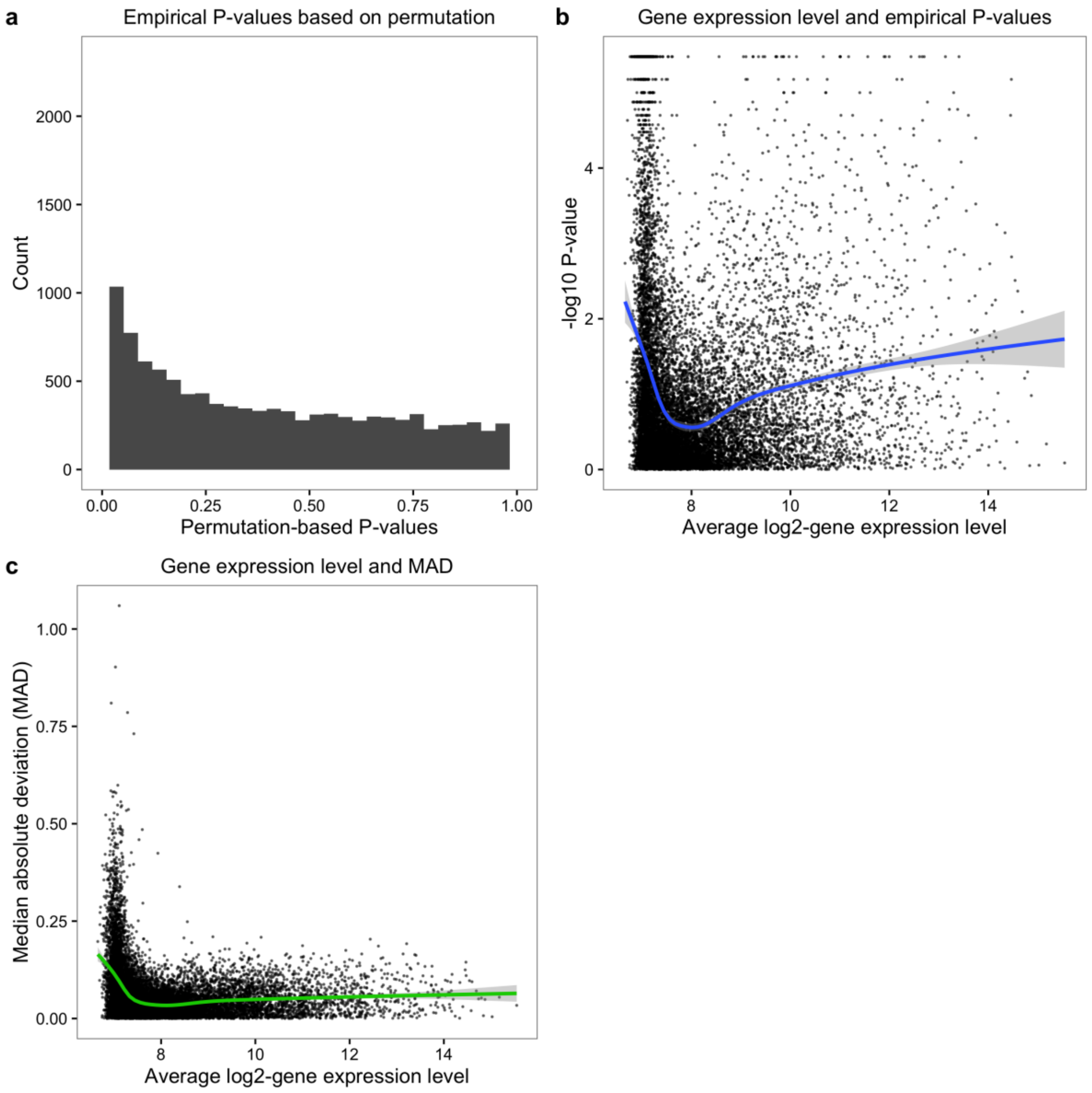

Supplementary Figure S6. Permutation-based *P*-value. (A) Histogram of empirical *P*-values based on 300,000 permutations. (B) -$\log_{10}$ empirical *P*-values are plotted against average gene expression levels. Blue line indicates the fitted relationship between -$\log_{10}$ *P*-values and average $\log_2$gene expression levels of cells that were detected as expressed, using locally weighted scatterplot smoothing (LOESS). (C) Median of Absolute Deviation (MAD) of genes versus average gene expression levels. Green line indicates the fitted relationship (LOESS) between the log10-MAD values and average $\log_2$gene expression levels of cells in which the gene was detected as expressed.

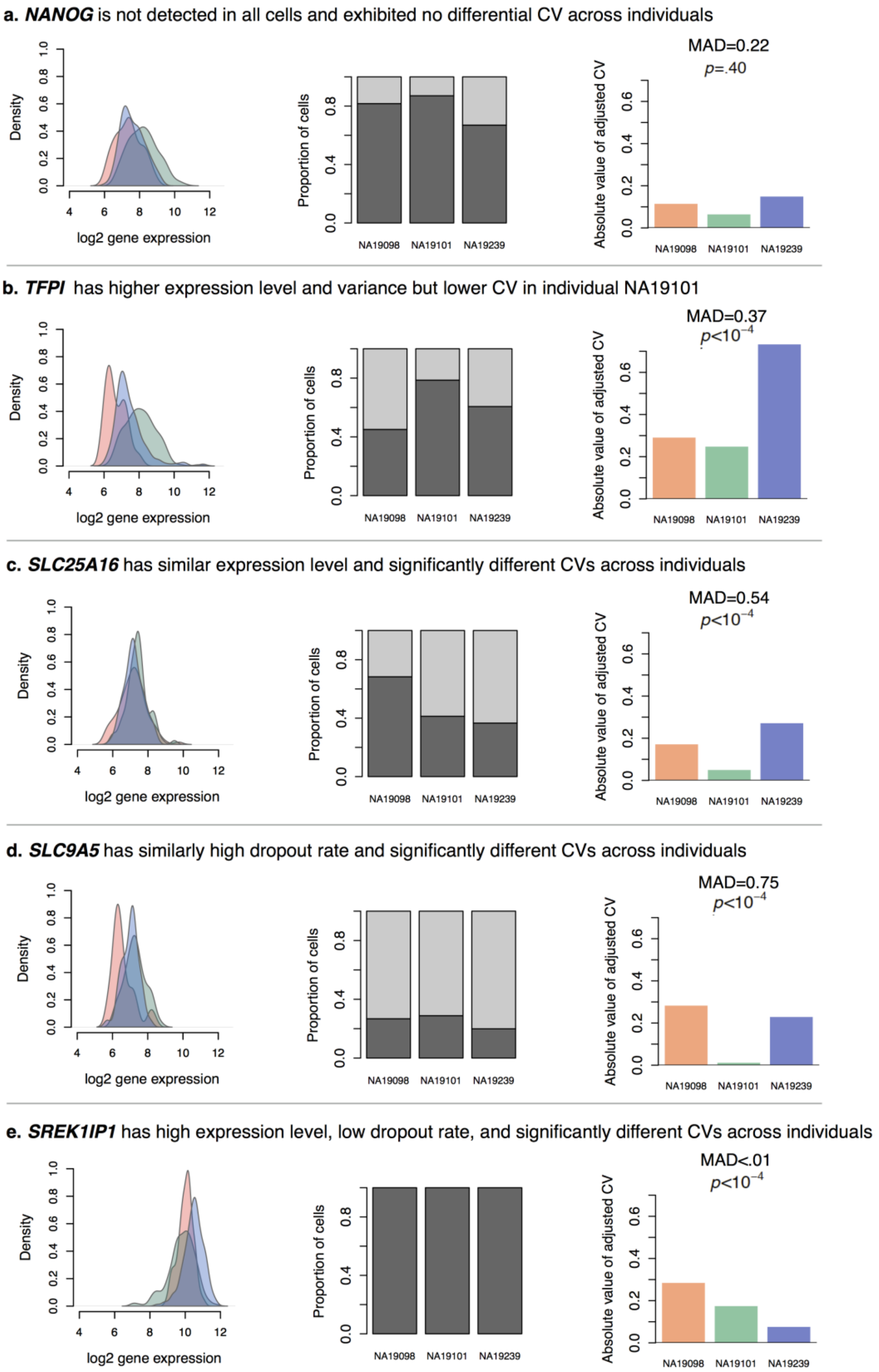

Supplementary Figure S7. Inter-individual differences in regulatory noise. These 5 example genes illustrate various patterns of cell-to-cell gene expression variance. For each

gene, the left panel shows the distribution of the $\log_2$ gene expression levels (considering only cells in which the gene is detected as expressed), the middle panel shows the proportion of cells in which the gene is detected as expressed (dark grey) and the dropout rate (light grey) for each individual, and the right panel shows the absolute value of adjusted CV for each individual, along with the corresponding gene-specific MAD (median of absolute deviation) value and *P*-value. The three colors in the upper and lower panel represent the individuals (NA19098 in red, NA19101 in green, and NA19239 in blue).

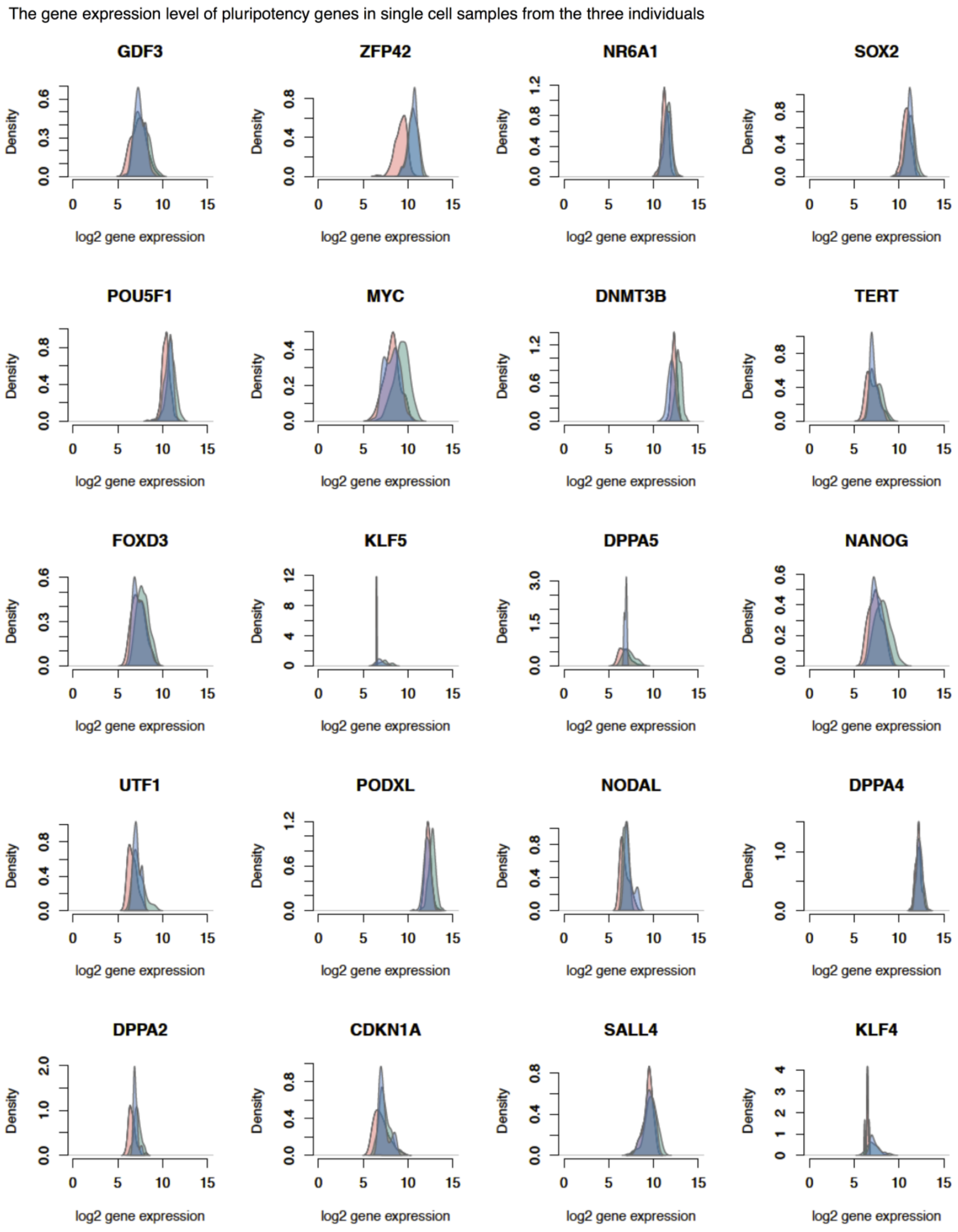

Supplementary Figure S8. Cell-to-cell variation of pluripotency genes. Density plots of the distribution of $\log_2$ gene expression of key pluripotency genes across all single cells by individual. The peaks with lower gene expression values (log2 around 4) represent the cells in

which the gene is undetected. The three colors represent the three individuals (NA19098 is in red, NA19101 in green, and NA19239 in blue).

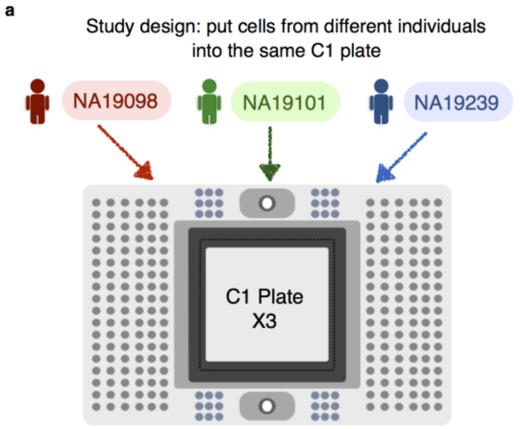

b

Results of identifying individuals based on the SNPs in RNA sequencing reads

Individual	Correct	Incorrect
19098	288	0
19101	288	0
19239	288	0

Supplementary Figure S9. Proposed study design for scRNA-seq using C1 platform. (A) A balanced study design consisting of multiple individuals within a C1 plate and multiple C1 replicates to fully capture the batch effect across C1 plates and further retrieve the maximum amount of biological information. (B) The correct identity of each single cell sample was determined by examining the SNPs present in their RNA sequencing reads.

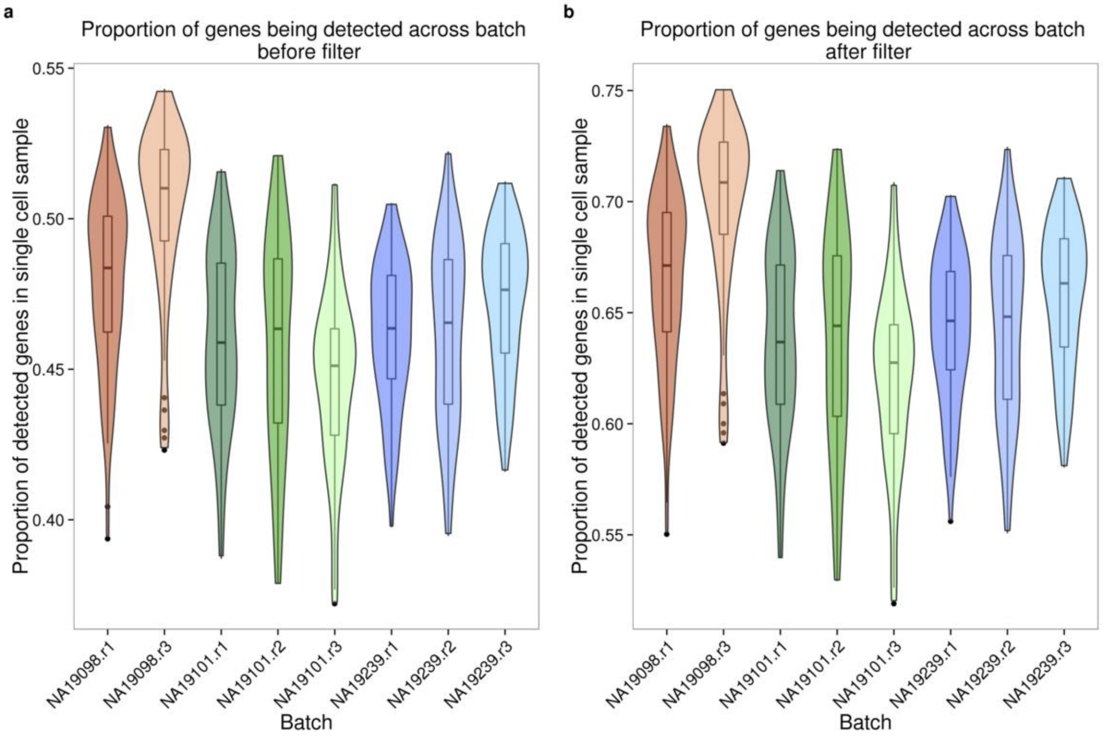

Supplementary Figure S10. The proportion of genes detected in single cell samples. Violin plots of the proportion of genes detected, computed by the total number of detected genes in each single cell divided by the total number of genes detected across all single cells,

before in (A) and after in (B) the removal of genes with low expression. The three colors represent the three individuals (NA19098 is in red, NA19101 in green, and NA19239 in blue).

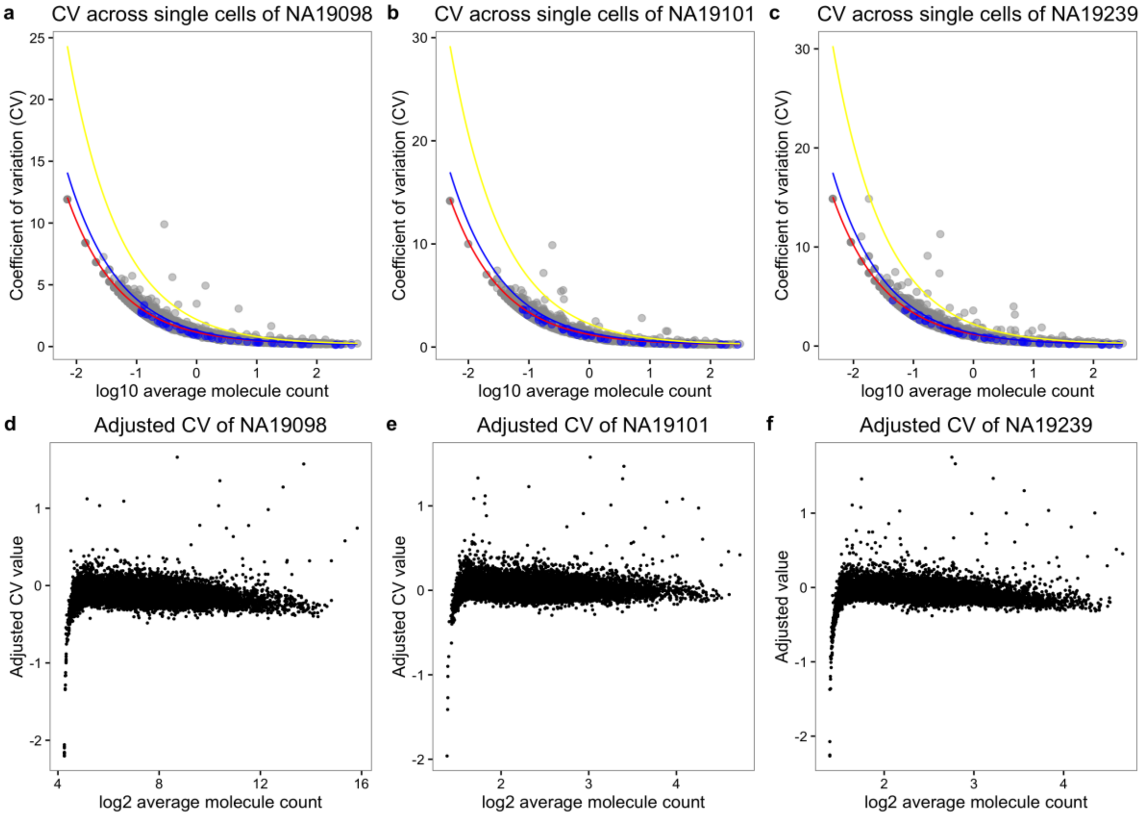

Supplementary Figure S11. Coefficients of variation (CV) before and after adjusting for gene mean abundance. (A-C) CV plotted against average molecule counts across all cells for each individual [20]. Grey points represent endogenous genes, and blue points represent ERCC spike-in controls. The curves indicate the expected CV under three different scenarios. Red curve depicts the expected CV of the endogenous genes while assuming a Poisson distribution with no over-dispersion. Likewise, blue curve depicts the expected CVs of the ERCC spike-in controls under the Poisson assumption. Yellow curve depicts the expected CVs of an over-dispersed Poisson distribution for which standard deviation is three times the ERCC spike-in controls. (D-F) Adjusted CV values of each gene including all cells are plotted against $\log_{10}$ of the average molecule counts for each individual.

Supplementary Tables

Supplementary Table S1. Data collection.

(A) iPSCs were sorted using the 10-17 m IFC plates with the staining of the pluripotency marker, TRA1-60. Single cell occupancy is the percentage of occupied capture sites containing one single cell. The average cDNA concentration was measured by the HT DNA high sensitivity LabChip (Caliper). (B) The 96 single cell libraries from one C1 plate were pooled and

sequenced in three HiSeq lanes. The pooled samples were assigned across the four 8-lane flowcells.

a Information and results of each C1 collections

Cell line	Passage	Input viability	No cell	Multiple cells	% single cell occupancy	TRA1-60 negative	% TRA1-60	Date	Ave cDNA con (ng/ul)	Replicate for seq
19098	12+15	89%	2	2	95.83	2	97.92	11/07/2014	N/A	(1)
19098	12+18	90%	2	4	93.75	0	100.00	11/14/2014	1.88	(2)
19098	12+20	83%	3	10	86.46	0	100.00	11/22/2014	1.40	(3)
19101	12+16	70%	2	3	94.79	9	90.63	11/13/2014	1.81	(1)
19101	12+19	94%	5	3	91.67	0	100.00	11/23/2014	1.38	(2)
19101	12+19	69%	1	19	79.17	0	100.00	11/24/2014	1.26	(3)
19239	12+16	85%	1	2	96.88	4	95.83	11/11/2014	1.60	(1)
19239	12+18	75%	1	6	92.71	5	94.79	11/17/2014	1.55	(2)
19239	12+19	93%	5	7	87.50	2	97.92	11/21/2014	1.70	(3)

b The arrangement of samples for sequencing on four flowcells

Flowcell 1	Flowcell 2	Flowcell 3	Flowcell 4
Bulk	19098 (2)	19098 (3)	19239 (1)
19098 (1)	19239 (3)	19101 (1)	19101 (2)
19239 (2)	19098 (1)	19098 (2)	19098 (3)
19101 (3)	19239 (2)	19239 (3)	19101 (1)
19239 (1)	19101 (3)	Bulk	19098 (2)
19101 (2)	19239 (1)	19098 (1)	19239 (3)
19098 (3)	19101 (2)	19239 (2)	Bulk (all 9)
19101 (1)	Bulk	19101 (3)	

Supplementary Table S2. High quality single cell samples.

List of the 564 high quality single cell samples.

Supplementary Table S3. Genes associated with inter-individual differences in regulatory noise.

List of genes that we classified the estimates of regulatory noise as significantly different across individuals (empirical permutation $P < 10^{-4}$). There are a total of 560 genes.

Supplementary Table S4. Gene ontology analysis of the genes associated with inter-individual differences in regulatory noise.
